# Supplementary material for: Damage activates EXG1 and RLP44 to suppress vascular differentiation during regeneration in Arabidopsis
Source: Plant Commun. 2025 Jan 16;6(4):101256. doi: 10.1016/j.xplc.2025.101256 (PMC12010363; doi:10.1016/j.xplc.2025.101256)
Supplement: Document S1. Figures S1–S6 and Tables S1–S4 [file mmc1.pdf]

Plant Communications, Volume *6*

Supplemental information

Damage activates *EXG1* and *RLP44* to suppress vascular differentiation during regeneration in *Arabidopsis*

Shamik Mazumdar, Frauke Augstein, Ai Zhang, Constance Musseau, Muhammad Shahzad Anjam, Peter Marhavy, and Charles W. Melnyk

Damage activates *EXG1* and *RLP44* to suppress vascular differentiation during regeneration in Arabidopsis

Shamik Mazumdar[1,a], Frauke Augstein[1], Ai Zhang[1,b], Constance Musseau[1,c], Muhammad Shahzad Anjam[2], Peter Marhavy[2], Charles W. Melnyk[1*]

1 - Department of Plant Biology, Linnean Center for Plant Biology, Swedish University of Agricultural Sciences, Almas allé 5, 756 51, Uppsala, Sweden

2 - Umea Plant Science Centre (UPSC), Department of Forest Genetics and Plant Physiology, Swedish University of Agricultural Sciences (SLU), 90183 Umea, Sweden

a - Present address: Department of Molecular Sciences, Linnean Center for Plant Biology, Swedish University of Agricultural Sciences, Almas allé 5, 756 51, Uppsala, Sweden

b - present address: College of Life Sciences, Northwest A&F University, No.3 Taicheng Road, Yangling, Shaanxi, China, 712100

c - Present address: Syngenta Seeds, Westeinde 62, 1601 BK Enkhuizen, Netherlands

*** Corresponding Author:** Charles W. Melnyk, email: charles.melnyk@slu.se, Department of Plant Biology, Linnean Center for Plant Biology, Swedish University of Agricultural Sciences, Almas allé 5, 756 51, Uppsala, Sweden

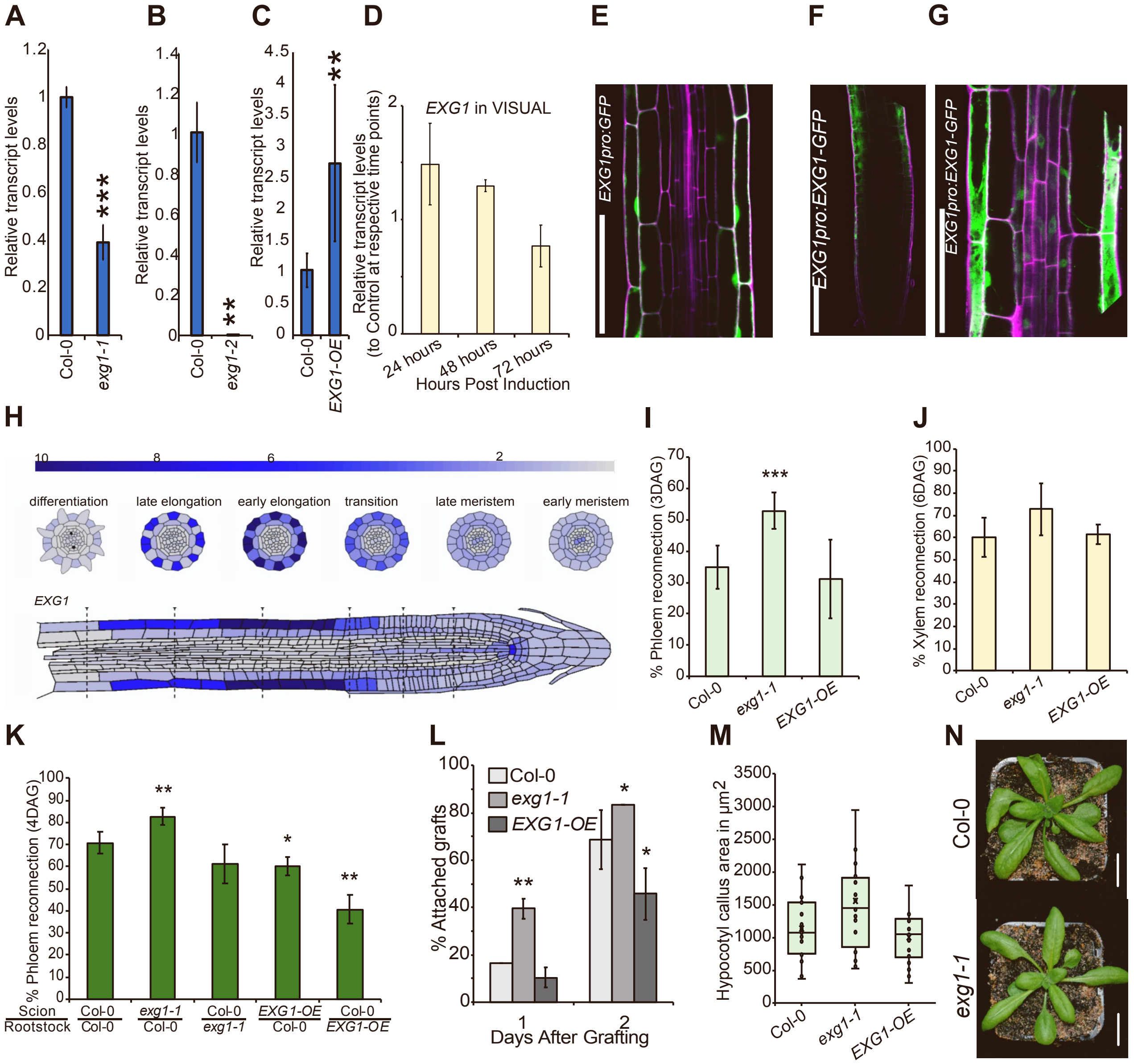

Supplemental Figure 1. *EXG1* expression levels, phenotypes. A-B. Expression of *EXG1* in *exg1-1* and *exg1-2* T-DNA insertion lines. C. Expression of *EXG1* in FOX hunting line *EXG1-OE*. D. Transcript levels of *EXG1* during VISUAL induction. E-F. *EXG1pro:GFP* fluorescence at the transition zone in the roots and at the root tip. G. *EXG1pro:EXG1-GFP* fluorescence at the transition zone in the roots. Cell walls stained by PI (magenta). Scale bar represents 100 µm. H. *EXG1* expression pattern in different cell layers of the primary root as generated from Root Cell Atlas (https://rootcellatlas.org/)Ryu et al., 2019; Denyer et al., 2019; Shulse et al., 2019; Jean-Baptiste et al., 2019; Wendrich et al., 2020; Shahan et al., 2022). I-J. Reconnection percentage of phloem (3 days after grafting – DAG) and xylem (6 DAG). The mean ± SD of 4-5 experiments is shown. K. Reconnection percentage of phloem (4DAG) during *EXG1* heterografting. The mean ± SD of 4 experiments is shown. L. Percentage of attached grafts at 1 DAG and 2 DAG. The mean ± SD of 4 experiments is shown. M. Hypocotyl explant callus areas of Col-0, *exg1-1* and *EXG1-OE*. Dots represent individual samples. N. Col-0 and exg1-1 plants in pots. Scale bar represents 1 cm. Asterisks indicate significant differences compared to Col-0. For transcript levels significance was calculate using Student's t-test ** p <0.01, *** p <0.001. For grafting and attachment assays significance was calculated by pairwise t-tests with Benjamini-Hochberg adjustment, *p<0.05, **p<0.01, ***p<0.001.

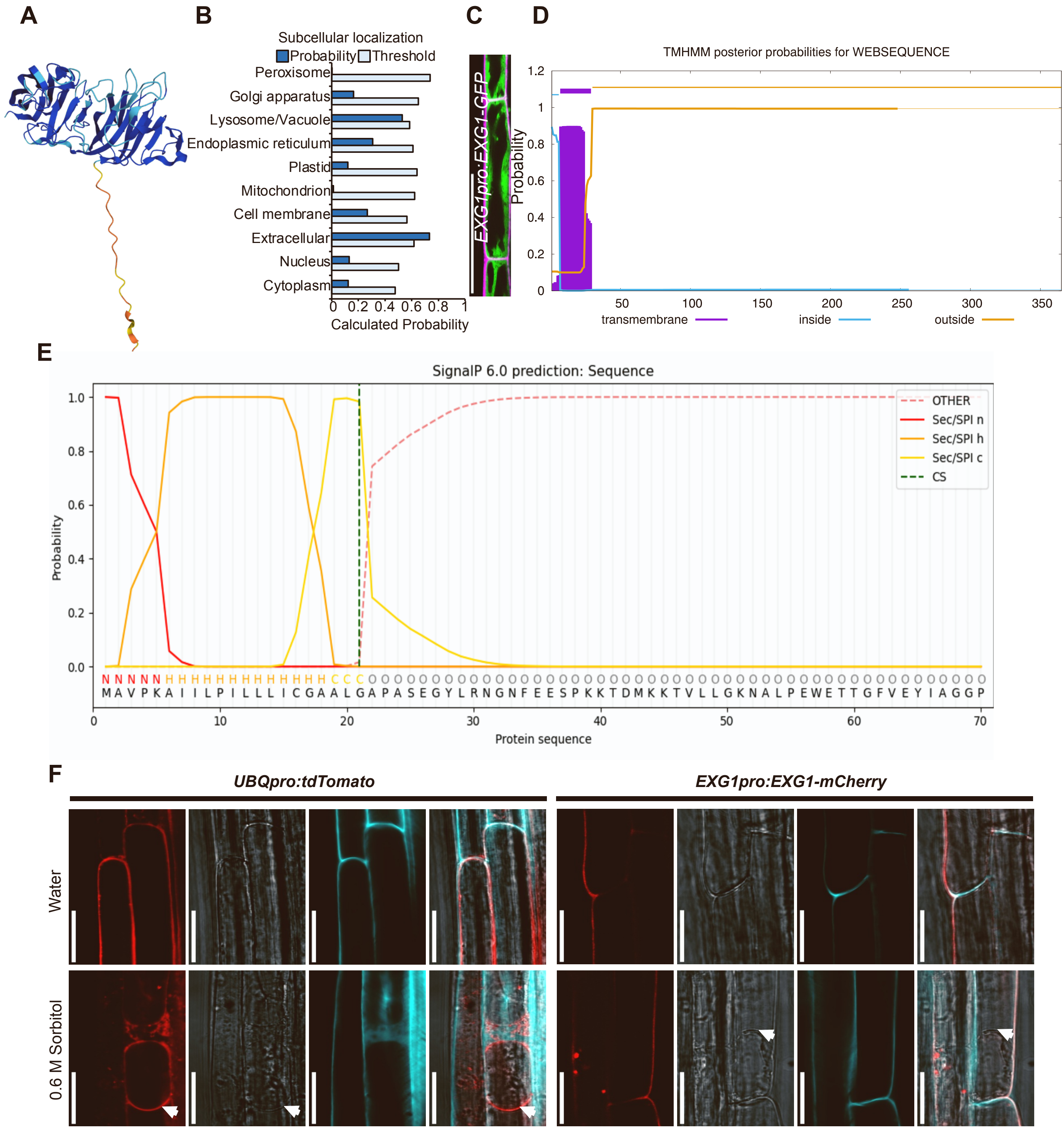

Supplemental Figure 2. *EXG1* protein predictions and subcellular localiations. A. Predicted protein structure of EXG1 using AlphaFold 2.0 (Jumper et al., 2021; Varadi et al., 2021). B. Threshold and calculated probability of subcellular localization of the EXG1 protein, predicted using DeepLoc2.0 (Thumuluri et al., 2022). C. *EXG1pro:EXG1-GFP* fluorescence in root tip epidermal cell. Cell walls stained by PI (magenta). Scale bar represents 50 µm. D. Transmembrane domain prediction using TMHMM (Krogh et al., 2001). E. Signal sequence prediction using Signal IP6.0 (Teufel et al., 2022). (F) Images showing plasmolysis of *EXG1pro:EXG1-mCherry* and *UBQpro:tdTomato* (in red), cell wall marked by Calcofluor white (cyan) in water and 0.6M sorbitol. White arrows indicate plasma membrane. Scale bars represent 20 µm.

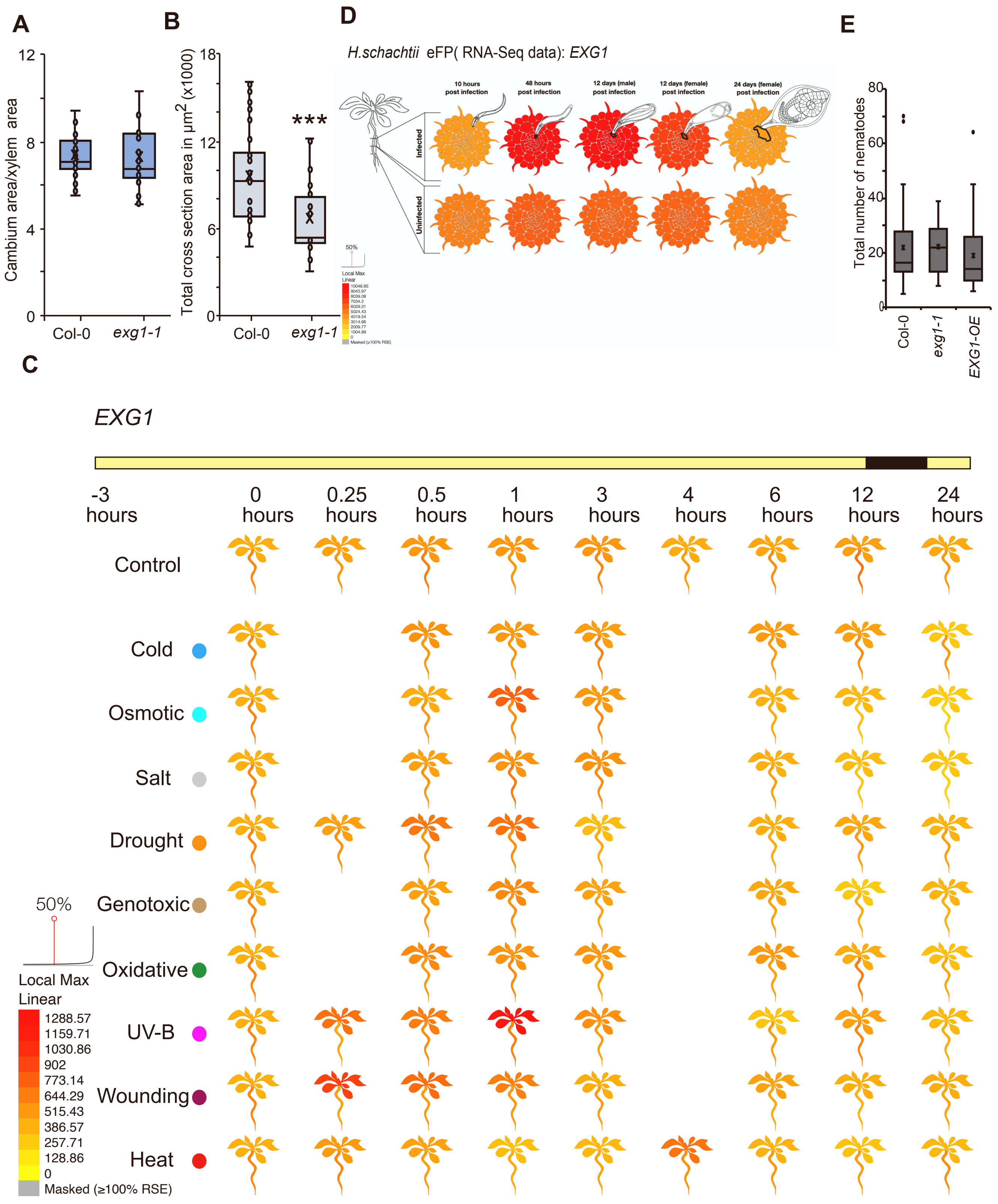

Supplemental Figure 3. *In silico* expression profiles of *EXG1* during stress, nematode infection. A. Cambium:xylem area quantifications. Dots represent individual samples. B. Total cross section area quantifications. Dots represent individual samples. C. *EXG1* expression during different abiotic stresses, eFP Browser profile (Kilian et al., 2007; Fucile et al., 2011). D. *EXG1* expression during *H. schachtii* infection. Data from ePlant (Fucile et al., 2011; Bar Toronto). E. Total number of nematodes per plant in Col-0, *exg1-1* and *EXG1-OE*. Dots indicate individual samples. Asterisks indicate statistical significance. For cross sections significance calculated by Wilcoxon's test, *** p<0.001.

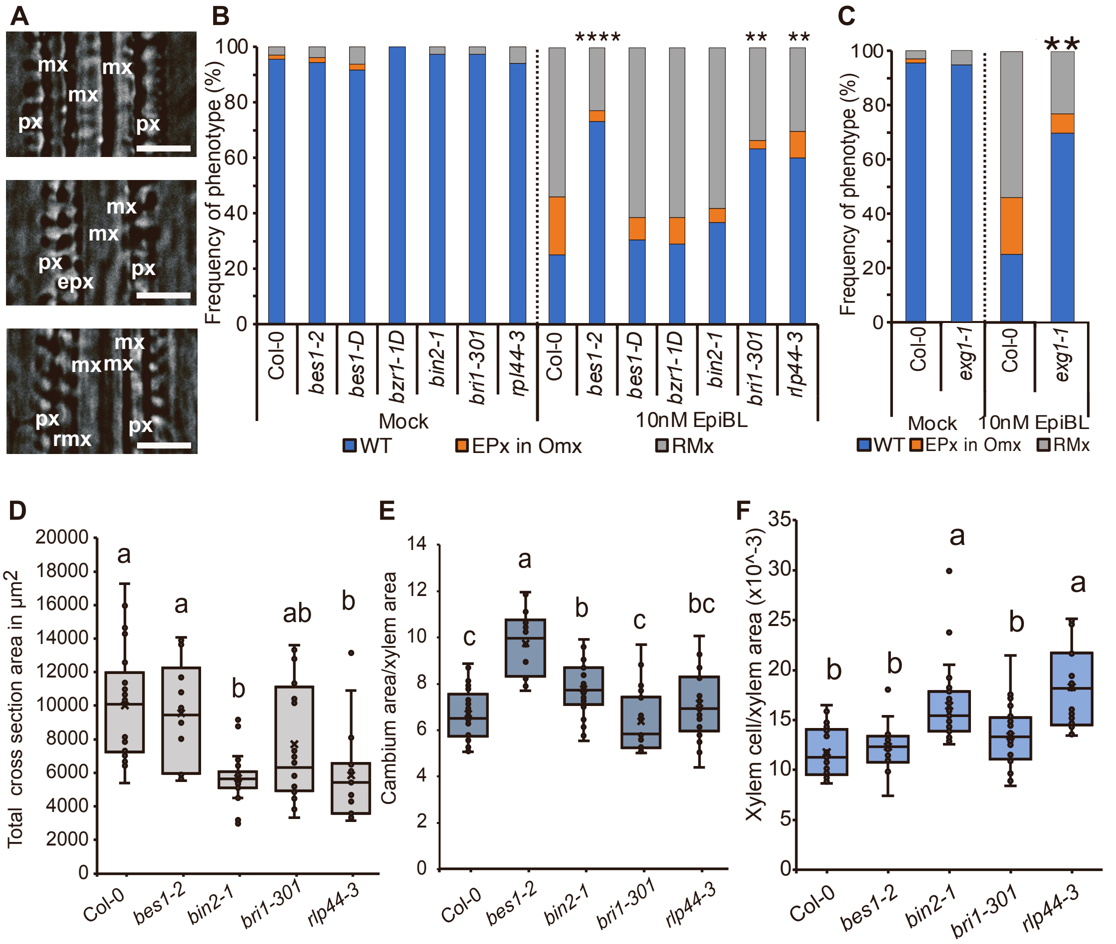

Supplemental Figure 4. Brassinosteroid mutants and *RLP44* in non-wounded conditions. A. Images showing primary root xylem phenotypes including protoxylem (px), metaxylem (mx), extra protoxylem (epx), reticulate metaxylem (rmx). Scale bar represents 200µM. B. Xylem phenotypes under mock and exogenous epiBL treatments: wild type (WT), extra protoxylem in outer metaxylem (EPx in Omx) or reticulate metaxylem (RMx) (n≥32). C. Xylem phenotypes of under mock and exogenous epiBL treatment: wild type (WT), extra protoxylem in outer metaxylem (EPx in Omx) or reticulate metaxylem (RMx) (n=50). D. Total cross section area comparisons. Dots represent samples. E. Cambium:xylem area comparisons. Dots represent samples. F. Xylem cell/ unit xylem area comparisons. Dots represent samples. Asterisks indicate statistical significance. For xylem phenotype significance was calculated by Fisher's exact test with Benjamini-Hochberg adjustment, **p<0.01, ****p<0.0001. For cross section significance was calculated by One-way ANOVA, with Tukey's post-hoc test. Compact letters indicate significant differences.

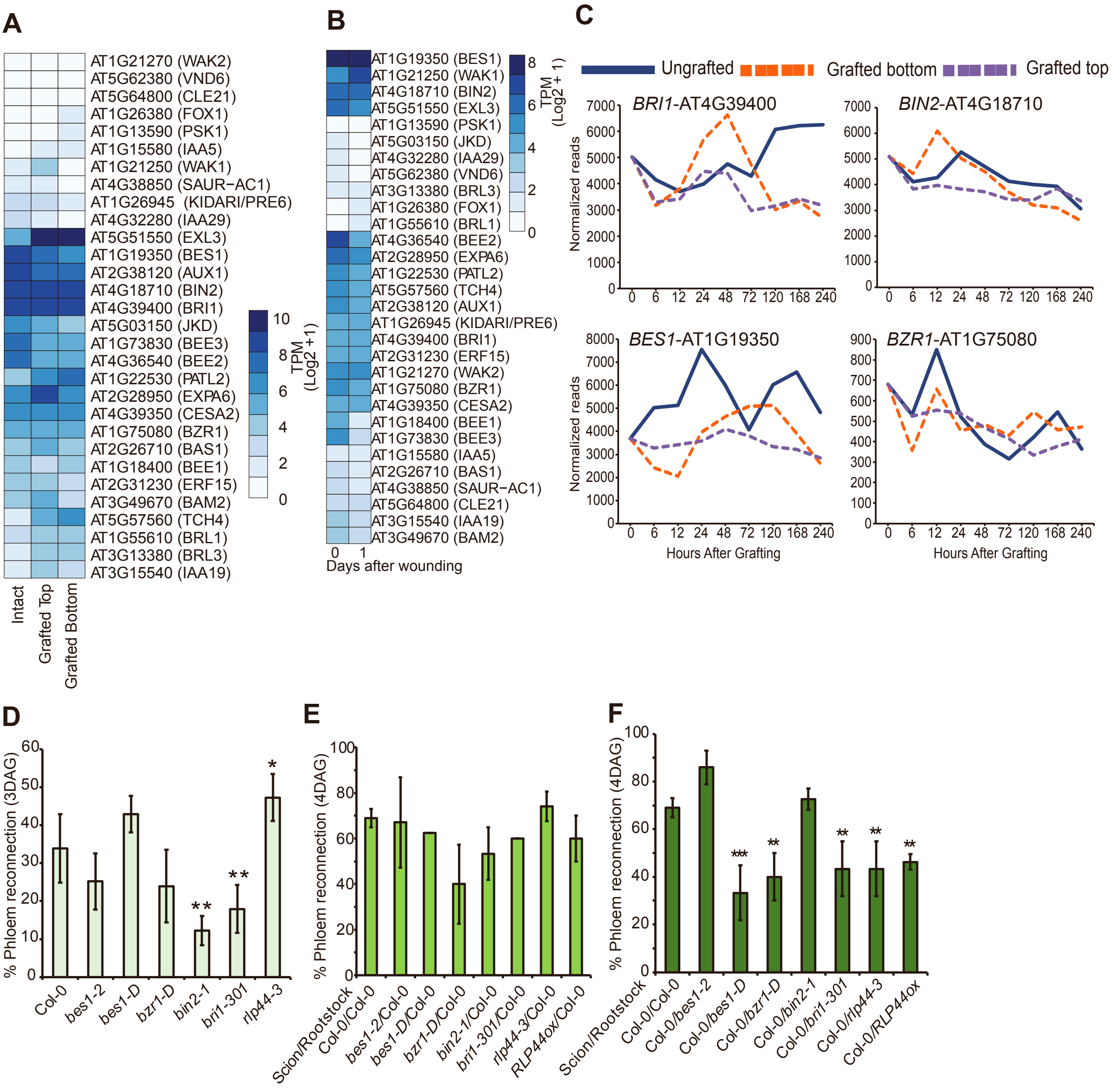

Supplemental Figure 5: BR responses and requirements at the graft junction. A. Heat map of the expression levels of brassinosteroid responsive genes at 24 hours after grafting (Zhang et al., 2022). B. Heat map of the expression levels of brassinosteroid responsive genes in petioles after wounding (Pan et al., 2019). C. Expression profile of brassinosteroid induced genes during graft formation (Melnyk et al. 2018). D. Reconnection percentage of phloem (3DAG). Mean ± SD of 4 experiments is shown. E-F. Reconnection percentage of phloem (4DAG) during heterografting. Mean ± SD of 3 experiments is shown. Asterisks indicate statistical significance. For grafting assays significance calculated by pairwise t-tests with Benjamini-Hochberg adjustment *p<0.05, ** p<0.01, *** p<0.001.

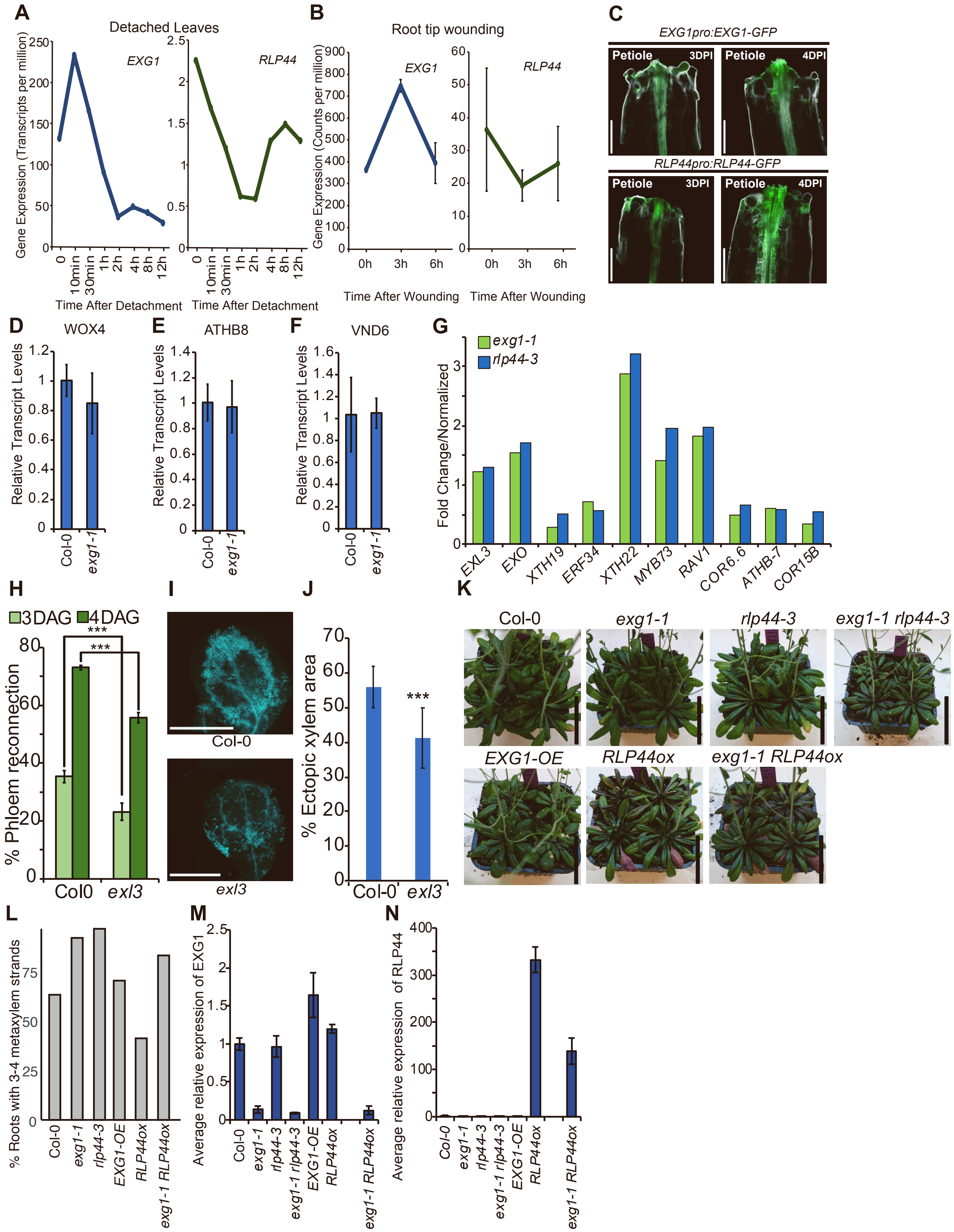

Supplemental Figure 6. *EXG1* is stress responsive and shares common downstream targets with *RLP44.* A. Expression of *EXG1* and *RLP44* in 12-day old, detached leaves of seedlings (Zhang et al., 2019) B. Expression of *EXG1* and *RLP44* in wounded root tips (Matosevich et al., 2020). C. *EXG1pro:EXG1-GFP* and *RLP44pro:RLP44-GFP* fusion protein fluorescence during petiole callus formation at 3 and 4 days post induction. Cell walls stained by Calcoflour white (grey). Scale bars represent 200 µm. D. Relative transcript levels of WOX4 in *exg1-1* compared to WT Col-0. E. Relative transcript levels of ATHB8 in *exg1-1* compared to WT Col-0. F. Relative transcript levels of VND6 in *exg1-1* compared to WT Col-0. G. Bar plot showing Fold change of a subset of genes differentially expressed in both *exg1-1* and *rlp44-3.* H. Reconnection percentage of phloem 3 and 4DAG. The mean ± SD of 3 experiments is shown. Asterisks indicate significant differences compared to Col-0. *** p<0.001, Student's t-test. I. VISUAL assay images of ectopic xylem formation. Scale bar represents 1 mm. J. % Ectopic xylem area quantifications. K. Images of mature plants. Scale bars represent 1cm. L. Metaxylem strand percentage. M. qPCR for average transcript level of EXG1. N. qPCR for average transcript level of RLP44. Asterisks indicate significant differences. For grafting assays significance was calculated using Student's t-test, ***p<0.001. For VISUAL assays significance was calculated using Wilcoxon's test, ***p<0.001.

Supplemental Table 1: List of lines used and generated in this study

AGI code	Name	Line	Mutant	Source	Donated by
AT3G08030	*EXG1*	*exg1-1*	SALK_011516C	This Study	NASC
AT3G08030	*EXG1*	*exg1-2*	SALK_119379C	This Study	NASC
AT3G08030	*EXG1*	*EXG1-OE*	-	(Ichikawa et al. 2006)	RIKEN BRC
AT3G08030	*EXG1*	*EXG1pro:GFP*	-	This Study	-
AT3G08030	*EXG1*	*EXG1pro:EXG1-GFP*		This Study	-
AT3G08030	*EXG1*	*EXG1pro:EXG1-mCherry*		This Study	-
-	-	*UBQpro:tdTomato*		(Segonzac et al. 2012)	Elliot Meyerowitz
AT3G49750	*RLP44*	*rlp44-3*	SAIL_596_E12	(Wolf et al. 2014)	Sebastian Wolf
AT3G49750	*RLP44*	*RLP44ox*	-	(Wolf et al. 2014)	
AT4G32880	*ATHB8*	*athb8-12*	-	(Prigge et al. 2005)	Annelie Carlsbecker
AT5G51550	*EXL3*	*exl3*	SALK_070475C	This Study	NASC
AT3G22600	*LTPG5*	*ltpg5*	SALK_007462C	(Edstam and Edqvist 2014)	NASC
AT5G10930	*CIPK5*	*cipk5*	SALK_063555C	(Förster et al. 2019)	NASC
AT4G24130	*SVB5*	*svb5*	SALK_006910C	(Hussain et al. 2021)	NASC
AT4G39400	*BRI1*	*bri1-301*		(Li and Nam 2002)	Sebastian Wolf
AT4G18710	*BIN2*	*bin2-1*		(Li et al. 2001)	
AT1G19350	*BES1*	*bes1-2*	WiscDsLox246D02	(Lachowiec et al. 2013)	Yuki Kondo
AT1G19350	*BES1*	*bes1-D*		(Yin et al. 2002)	
AT1G75080	*BZR1*	*bzr1-D*		(Wang et al. 2002)	

Supplemental Table 2: Primers used for genotyping

Name	Sequence	Reason
EXG1-F1	CAAAGACGACAAAATTCCCAC	Genotyping *EXG1-1*
EXG1-R1	CGTGAGTGCGTAGAGAGAACC	
EXG1-F2	CACTAACCAAGGAAAACTCATTCTC	Genotyping *EXG1-2*
EXG1-R2	CAAGCTCTTTAATGGCGACAG	
Exl3-F1	GCAATCATTAGGCAAAAGCTG	Genotyping *exl3*
Exl3-R1	TCAGCCGTTGGATCTAAACAC	
CIPK5-F1	GGAAAGTACGAGATGGGAAGG	Genotyping *cipk5*
CIPK5-R1	TGTTCATCTCCTTCGTAACCG	
LTPG5-F1	TGGGATGCGTACATGTGTATG	Genotyping *ltpg5*
LTPG5-R1	ATGTAGTTGAGACACGGCGAC	
SVB5-F1	TTGCTTCATCCAAACGTAACC	Genotyping *svb5*
SVB5-R1	AAGGGCTTTGGACTTTACCTG	
RLP44-3-F1	ATTCCACTCCCAAGTCCAATC	Genotyping *rlp44-3*
RLP44-3-R1	AATGGATGGCATGATTAGGATC	
SALK_LB1	ATTTTGCCGATTTCGGAAC	LBb1.3 SALK pROK2
SAIL_LB1	GCCTTTTCAGAAATGGATAAATAGCCTTGCTTCC	LB1 for SAIL lines C/418-451 of pCSA110-pDAP101_T-DNAs
pDs-Lox_LB1	AACGTCCGCAATGTGTTATTAAGTTGTC	WiscDsLox T-DNA LB primer P745

Supplemental Table 3: Primers used for expression analysis

Name	Sequence	Reason
EXG1_qP-F	GCTCCTGCTTCTGAAGGTTATC	qPCR primers for EXG1
EXG1_qP-R	AACCGGTGGTTTCCCATTCG	
EXL3-qP-F1	CAAGATTTTTGCGGCCAGGT	qPCR primers for EXL3
EXL3-qP-R1	AAGCACAAACTCCGGGACAA	
CIPK5-qP-F1	CGCCGAAATTCTTCAACGCT	qPCR primers for CIPK5
CIPK5-qP-R1	GACCGTGACGTAAACACCGA	
LTPG5-qP-F1	CTTGGTCTGCCTAGGGCTTG	qPCR primers for LTPG5
LTPG5-qP-R1	CCTGGTCCTGAAGAGTTTGG	
SVB5-qP-F1	ATGACAGGAGTGAAGAGTAAGCAG	qPCR primers for SVB5
SVB5-qP-R1	CGGTAACGGGGAAAGACCTG	

Supplemental Table 4: Primers used for Cloning

Name	Sequence	Cloning module	Reason
EXG1-proF	AACAGGTCTCAACCTCACCGATGGTGACATTG	Greengate	*EXG1* promoter
EXG1-proR	AACAGGTCTCATGTTTGTCTCTGTTGTTCTTCC	Greengate	
RbcsT-F	AACAGGTCTCACTGCAGAGCTTTCGTTCGTATC	Greengate	Pea Rbcs9-E terminator
RbcsT-R	ACAAGGTCTCATAGTGTTGTCAATCAATTGGC	Greengate	
GFP-CDS-F	AACAGGTCTCAGGCTATGGTGAGCAAGGGCG	Greengate	GFP coding sequence
GFP-CDS-R	ACAAGGTCTCACTGACTACTTGTACAGCTCGTCC	Greengate	

References

Edstam MM and Edqvist J. Involvement of GPI-anchored lipid transfer proteins in the development of seed coats and pollen in *Arabidopsis thaliana*. Physiol Plantarum. 2014:**152**(1):32–42. https://doi.org/10.1111/ppl.12156

Förster S, Schmidt LK, Kopic E, Anschütz U, Huang S, Schlücking K, Köster P, Waadt R, Larrieu A, Batistič O, et al. Wounding-Induced Stomatal Closure Requires Jasmonate-Mediated Activation of GORK K+ Channels by a Ca2+ Sensor-Kinase CBL1-CIPK5 Complex. Developmental Cell. 2019:**48**(1):87-99.e6. https://doi.org/10.1016/j.devcel.2018.11.014

Hussain S, Zhang N, Wang W, Ahmed S, Cheng Y, Chen S, Wang X, Wang Y, Hu X, Wang T, et al. Involvement of ABA Responsive SVB Genes in the Regulation of Trichome Formation in Arabidopsis. IJMS. 2021:**22**(13):6790. https://doi.org/10.3390/ijms22136790

Ichikawa T, Nakazawa M, Kawashima M, Iizumi H, Kuroda H, Kondou Y, Tsuhara Y, Suzuki K, Ishikawa A, Seki M, et al. The FOX hunting system: an alternative gain-of-function gene hunting technique. The Plant Journal. 2006:**48**(6):974–985. https://doi.org/10.1111/j.1365-313X.2006.02924.x

Lachowiec J, Lemus T, Thomas JH, Murphy PJM, Nemhauser JL, and Queitsch C. The Protein Chaperone HSP90 Can Facilitate the Divergence of Gene Duplicates. Genetics. 2013:**193**(4):1269–1277. https://doi.org/10.1534/genetics.112.148098

Li J and Nam KH. Regulation of Brassinosteroid Signaling by a GSK3/SHAGGY-Like Kinase. Science. 2002:**295**(5558):1299–1301. https://doi.org/10.1126/science.1065769

Li J, Nam KH, Vafeados D, and Chory J. *BIN2* , a New Brassinosteroid-Insensitive Locus in Arabidopsis. Plant Physiology. 2001:**127**(1):14–22. https://doi.org/10.1104/pp.127.1.14

Prigge MJ, Otsuga D, Alonso JM, Ecker JR, Drews GN, and Clark SE. Class III Homeodomain-Leucine Zipper Gene Family Members Have Overlapping, Antagonistic, and Distinct Roles in Arabidopsis Development. The Plant Cell. 2005:**17**(1):61–76. https://doi.org/10.1105/tpc.104.026161

Segonzac C, Nimchuk ZL, Beck M, Tarr PT, Robatzek S, Meyerowitz EM, and Zipfel C. The Shoot Apical Meristem Regulatory Peptide CLV3 Does Not Activate Innate Immunity. Plant Cell. 2012:**24**(8):3186–3192. https://doi.org/10.1105/tpc.111.091264

Wang Z-Y, Nakano T, Gendron J, He J, Chen M, Vafeados D, Yang Y, Fujioka S, Yoshida S, Asami T, et al. Nuclear-Localized BZR1 Mediates Brassinosteroid-Induced Growth and Feedback Suppression of Brassinosteroid Biosynthesis. Developmental Cell. 2002:**2**(4):505–513. https://doi.org/10.1016/S1534-5807(02)00153-3

Wolf S, van der Does D, Ladwig F, Sticht C, Kolbeck A, Schürholz A-K, Augustin S, Keinath N, Rausch T, Greiner S, et al. A receptor-like protein mediates the response to pectin modification by activating brassinosteroid signaling. Proc Natl Acad Sci USA. 2014:**111**(42):15261–15266. https://doi.org/10.1073/pnas.1322979111

Yin Y, Wang Z-Y, Mora-Garcia S, Li J, Yoshida S, Asami T, and Chory J. BES1 Accumulates in the Nucleus in Response to Brassinosteroids to Regulate Gene Expression and Promote Stem Elongation. Cell. 2002:**109**(2):181–191. https://doi.org/10.1016/S0092-8674(02)00721-3
