# Supplementary material for: Damage activates EXG1 and RLP44 to suppress vascular differentiation during regeneration in Arabidopsis
Source: Plant Commun. 2025 Jan 16;6(4):101256. doi: 10.1016/j.xplc.2025.101256 (PMC12010363; doi:10.1016/j.xplc.2025.101256)
Supplement: Document S3. Article plus supplemental information [file mmc3.pdf]

Plant Communications
Research article

CellPress Partner Journal

Damage activates *EXG1* and *RLP44* to suppress vascular differentiation during regeneration in *Arabidopsis*

Shamik Mazumdar[1,3], Frauke Augstein[1], Ai Zhang[1,4], Constance Musseau[1,5], Muhammad Shahzad Anjam[2], Peter Marhavy[2] and Charles W. Melnyk[1,*]

[1]Department of Plant Biology, Linnean Center for Plant Biology, Swedish University of Agricultural Sciences, Almas Allé 5, 756 51 Uppsala, Sweden

[2]Umea Plant Science Centre (UPSC), Department of Forest Genetics and Plant Physiology, Swedish University of Agricultural Sciences (SLU), 901 83 Umea, Sweden

[3]Present address: Department of Molecular Sciences, Linnean Center for Plant Biology, Swedish University of Agricultural Sciences, Almas Allé 5, 756 51 Uppsala, Sweden

[4]Present address: College of Life Sciences, Northwest A&F University, No. 3 Taicheng Road, Yangling 712100, Shaanxi, China

[5]Present address: Syngenta Seeds, Westeinde 62, 1601 BK Enkhuizen, Netherlands

***Correspondence: Charles W. Melnyk (charles.melnyk@slu.se)**

https://doi.org/10.1016/j.xplc.2025.101256

ABSTRACT

Plants possess remarkable regenerative abilities to form *de novo* vasculature after damage and in response to pathogens that invade and withdraw nutrients. To identify common factors that affect vascular formation upon stress, we searched for *Arabidopsis thaliana* genes differentially expressed upon *Agrobacterium* infection, nematode infection, and plant grafting. One such gene is cell wall-related and highly induced by all three stresses, which we named *ENHANCED XYLEM AND GRAFTING1* (*EXG1*), since its mutations promote ectopic xylem formation in a vascular cell induction system and enhance graft formation. Further observations revealed that *exg1* mutants show inhibited cambium development and callus formation but enhanced tissue attachment, syncytium size, phloem reconnection, and xylem formation. Given that brassinosteroids also promote xylem differentiation, we analyzed brassinosteroid-related genes and found that mutations in *RLP44* encoding a receptor-like protein cause similar regeneration-related phenotypes as mutations in *EXG1*. Like *EXG1*, *RLP44* expression is also induced by grafting and wounding. Mutations in *EXG1* and *RLP44* affect the expression of many genes in common, including those related to cell walls and genes important for vascular regeneration. Our results suggest that *EXG1* integrates information from wounding or pathogen stress and functions with *RLP44* to suppress vascular differentiation during regeneration and healing.

Key words: wounding, xylem, grafting, cell wall, regeneration, stress

Mazumdar S., Augstein F., Zhang A., Musseau C., Anjam M.S., Marhavy P., and Melnyk C.W. (2025). Damage activates *EXG1* and *RLP44* to suppress vascular differentiation during regeneration in *Arabidopsis*. Plant Comm. **6**, 101256.

INTRODUCTION

The ability of plants to regenerate tissues after injury is of fundamental importance to maintain tissue integrity and promote regrowth. Upon wounding, plants activate defense and regeneration responses to deter further injury and heal damage. Wounding induces cell-wall damage, causes auxin accumulation, and increases auxin response around the injury. These processes activate several transcriptional factors such as *ETHYLENE RESPONSE FACTOR* (*ERF115*), *TARGET OF MONOPTEROS 6* (*TMO6*), and *HIGH CAMBIAL ACTIVITY* (*HCA2*) that are important for wound healing (Canher et al., 2020; Hoermayer et al., 2020; Zhang et al., 2022). During early stages of regeneration, cells close to the wound site expand and deposit cell-wall materials such as pectin, which can help tissues adhere (Sala et al., 2019; Zhang et al., 2022). Cells dedifferentiate and divide to form a mass of pluripotent stem cells known as callus, which are regulated by cell-cycle genes including *CYCLIN D3;1* (*CYCD3;1*) and the transcription factor *WOUND INDUCED DIFFERENTIATION1* (*WIND1*) that activates cytokinin responses (Iwase et al., 2011a; Ikeuchi et al., 2017). Callus tissues fill the wound and differentiate to reform

© 2025 The Authors. Published by Elsevier Inc. on behalf of CAS Center for Excellence in Molecular Plant Sciences, Chinese Academy of Sciences, and Chinese Society for Plant Biology. This is an open access article under the CC BY license (http://creativecommons.org/licenses/by/4.0/).

missing cell types and reconnect vasculature by forming new phloem and xylem (Iwase et al., 2021).

Similar recognition and healing processes occur during plant grafting, a horticulturally relevant technique, when two plants are cut and joined to create a new plant. At the graft junction, thousands of genes are differentially expressed, with cambium-related genes activating first, followed by phloem-related and then xylem-related genes (Melnyk et al., 2018). Several genes have been identified as important for graft formation, including the auxin-related genes *ABERRANT LATERAL ROOT FORMATION 4* (*ALF4*) and *AUXIN RESISTANT 1* (*AXR1*) that are needed below the graft junction, and cambium-related genes such as *HCA2*, *TMO6*, *WUSCHEL-RELATED HOMEOBOX4* (*WOX4*), and *NAC DOMAIN-CONTAINING PROTEIN 96* (*NAC0969*) (Melnyk et al., 2015, 2018; Matsuoka et al., 2016; Thomas et al., 2022; Zhang et al., 2022). Mutations in these genes reduce vascular connectivity or cambium formation; however , no recessive mutations that improve grafting have been identified to date.

Given the importance of cell walls during regeneration, it is surprising that during graft healing no role has been found for brassinosteroids (Nanda and Melnyk, 2018), a group of plant hormones involved in vascular development and maintaining cell-wall homeostasis (Caño-Delgado et al., 2004; Ibañes et al., 2009; Wolf et al., 2012; Lozano-Elena and Caño-Delgado, 2019; Oh et al., 2020). Activating brassinosteroid signaling is critical for forming ectopic xylem from leaf mesophyll cells using the vascular cell induction culture system using *Arabidopsis* leaves (VISUAL) system (Kondo et al., 2015). By enhancing or suppressing brassinosteroid signaling, more or less ectopic xylem is formed (Kondo et al., 2015). However, during normal root development, mutations in the genes encoding brassinosteroid receptors including BRASSINOSTEROID INSENSITIVE 1 (BRI1) promote xylem formation and suppress cambium (Kang et al., 2017), yet this role of BRI1 seems independent of canonical brassinosteroid signaling and instead is related to its interaction with RECEPTOR-LIKE PROTEIN 44 (RLP44) (Holzwart et al., 2018). During root development, BRI1–RLP44 associates with the phytosulfokine pathway to promote cambial identity but repress xylem differentiation (Holzwart et al., 2018). Phytosulfokine signaling is known to be activated by wound-induced *ERF115* (Heyman et al., 2013). However, the role of canonical and non-canonical brassinosteroid signaling during grafting and regeneration remains poorly characterized.

Although most pathogens induce plant defense responses, some can activate the regeneration pathways to infect their hosts more efficiently. *Agrobacterium* enters plant wound sites and induces auxin and cytokinin production to cause cell differentiation, cell division, vascularization, and tumor growth (Deeken et al., 2007; Zhang et al., 2015). Root-knot nematodes and cyst nematodes feed on plant roots and cause cell proliferation to derive nutrients from host plants (Shanks et al., 2016; Olmo et al., 2020). They also activate host genes that regulate vascular development, such as *HOMEOBOX-8* (*ATHB8*), *WOX4*, and *TRACHEARY ELEMENT DIFFERENTIATION INHIBITORY FACTOR RECEPTOR* (*TDR/PXY*) (Yamaguchi et al., 2017). The cyst nematode *Heterodera schachtii* releases CLE-like effector proteins into plant cells to induce cell proliferation and activate the *WOX4*-mediated cambium-promoting pathway (Guo et al., 2017). Thus, *WOX4* activation during both nematode infection and grafting suggests an overlap in common processes induced during various forms of regeneration or parasitism (Melnyk, 2017d). However, what genes regulate these common processes and how they promote or inhibit parasitism and regeneration remain largely unknown. In this study, we investigated these aspects and identified and characterized a gene, AT3G08030, also known as *ATHA2-1* due to its phylogenetic relatedness to a clade of DUF642 protein genes (Vázquez-Lobo et al., 2012). Mutants of *AT3G08030* exhibit enhanced vascular formation in grafting and VISUAL assays, and increased xylem formation in primary roots, leading us to name this gene *ENHANCED XYLEM AND GRAFTING 1* (*EXG1*). This gene regulates multiple regeneration and developmental processes, and its mutants appeared phenotypically similar to mutants of *RLP44* in affecting development and regeneration. Given that *EXG1* is highly and rapidly activated upon wounding, we propose that it acts as a stress-responsive gene that functions with *RLP44* to balance cambial proliferation and vascular differentiation.

RESULTS

EXG1 is stress-responsive and regulates regeneration post wounding

To identify genes differentially expressed in response to stress, we compared previously published *Arabidopsis thaliana* transcriptomic datasets related to *Agrobacterium* infection, nematode infection, and plant grafting (Deeken et al., 2007; Szakasits et al., 2009; Barcala et al., 2010; Melnyk et al., 2018). Of those differentially expressed in at least two datasets, we selected genes associated with vascular development and narrowed our list to 22 candidates, which include previously described vascular-related genes such as *ATHB8* (AT4G32880) and *WRKY23* (AT2G47260) (Baima et al., 2001; Prát et al., 2018) (Figure 1A). As a second method to find novel vascular regulators, we employed the VISUAL system of ectopic xylem formation, which can rapidly identify mutants related to vascular development (Kondo et al., 2015). Transfer-DNA (T-DNA) mutant lines were tested using VISUAL, and many showed reduced ectopic xylem formation, consistent with a role for their corresponding genes in promoting vascular development (Figure 1B). However, AT3G08030/*EXG1* appeared exceptional, since its mutants of this gene exhibited increased levels of ectopic xylem formation (Figure 1B). We obtained a second T-DNA mutant line of *EXG1* named *exg1-2* and an overexpression line, *35Spro:EXG1-cDNA* (*EXG1-OE*), from the FOX hunting system (Ichikawa et al., 2006), and confirmed the relative transcript levels of *EXG1* in the respective lines (Supplemental Figure 1A–1C). In subsequent VISUAL assays for xylem formation, *exg1-2* showed enhanced ectopic xylem formation, like *exg1-1*, whereas *EXG1-OE* exhibited reduced ectopic xylem formation compared with the wild-type Columbia-0 (Col-0) (Figure 1C and 1D). During VISUAL, (pro)cambium-related gene expression peaks at 24 h post induction and subsequently decreases at later time points (Kondo et al., 2015). We found that *EXG1* transcript levels were also elevated at 24 h and reduced as time progressed (Supplemental Figure 1D). We generated transcriptional and translational reporters to further understand spatiotemporal dynamics of *EXG1* expression. In *Arabidopsis* primary roots, transcriptional reporters showed highest fluorescence in the root

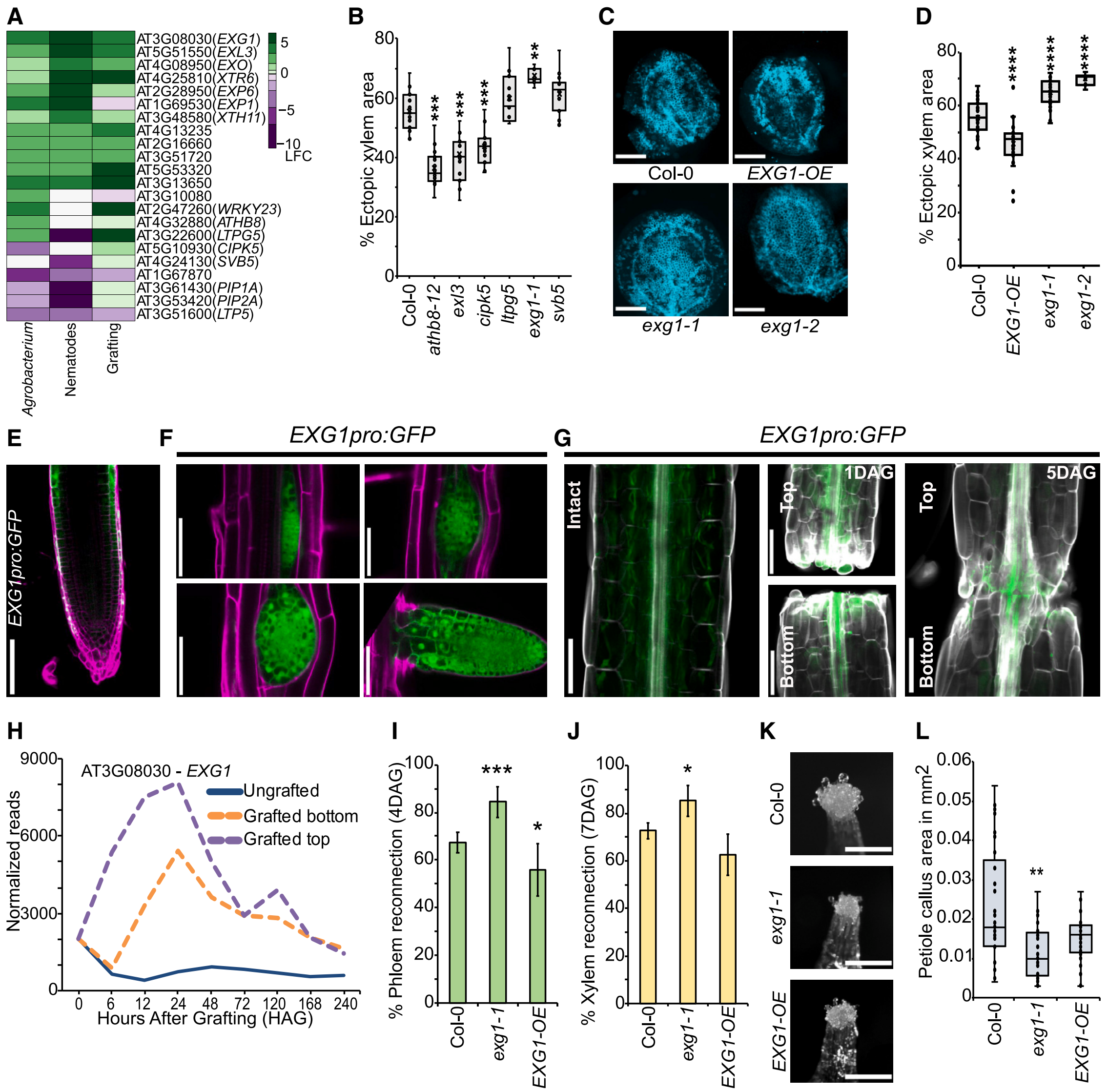

Figure 1. *EXG1* is activated by stress and affects regeneration in VISUAL and grafting assays.

(A) Heatmap showing differentially expressed genes in *Agrobacterium*-infected hosts (Deeken et al., 2007), nematode-infected hosts (Szakasits et al., 2009; Barcala et al., 2010), and grafted top versus ungrafted at 24 h (Melnyk et al., 2018).

(B) VISUAL assay quantification of percentage ectopic xylem area. Dots represent individual samples.

(C) VISUAL assay images of ectopic xylem formation in *EXG1* mutants. Scale bars represent 1 mm.

(D) Percentage ectopic xylem area quantification. Dots represent individual samples.

(E) *EXG1pro:GFP* fluorescence at the root tip. Cell walls were stained by PI (magenta). Scale bar represents 100 μm.

(F) *EXG1pro:GFP* fluorescence in different stages of lateral root development. Cell walls were stained by PI (magenta). Scale bars represent 100 μm.

(G) *EXG1pro:GFP* (green) during graft formation. Comparison of intact, grafted top, and grafted bottom 1 and 5 days after grafting (DAG) . Cell walls were stained by Calcofluor white (gray). Scale bars represent 100 μm.

(H) Dynamics of *EXG1* expression during grafting (Melnyk et al., 2018).

(I and J) Reconnection percentage of phloem (4 DAG) and xylem (7 DAG). The mean ± SD of five experiments is shown.

(K) Representative images of callus formation in petiole explants. Scale bars represent 250 μm.

(L) Petiole callus area quantification.

Asterisks indicate statistical significance compared to Col-0. For VISUAL and petiole callus assays significance was calculated by Wilcoxon's test: $^{**}p < 0.01$, $^{***}p < 0.001$, and $^{****}p < 0.0001$. For grafting assays significance was calculated by pairwise *t*-tests with Benjamini–Hochberg adjustment: $^{*}p < 0.05$ and $^{**}p < 0.01$.

epidermis, with little signal in the cortex or stele (Figure 1E; Supplemental Figure 1E–1G). Previously published datasets confirmed the expression of *EXG1* in outer cell layers of the root (Supplemental Figure 1H) (https://rootcellatlas.org/) (Denyer et al., 2019; Jean-Baptiste et al., 2019; Ryu et al., 2019; Shulse et al., 2019; Zhang et al., 2019; Wendrich et al., 2020; Shahan et al., 2022). However, in lateral root primordia, we observed strong *EXG1* expression throughout the inner and outer cell layers (Figure 1F). We tested the *EXG1* transcriptional reporter during graft formation, and found that the signal increased

at the cut site and in vascular tissues compared with unwounded intact plants (Figure 1G). Moreover, we found the signal in both the grafted top and the grafted bottom at 1 day after grafting (1 DAG) and at the graft junction at 5 DAG, consistent with *EXG1* transcriptional dynamics during graft formation from a previously published dataset (Melnyk et al., 2018) (Figure 1G and 1H). To test the role of *EXG1* during graft formation, we performed *Arabidopsis* hypocotyl grafting experiments. We applied the vascular mobile dye carboxyfluorescein diacetate (CFDA) to grafted scions or rootstocks using previously described assays (Melnyk et al., 2015; Melnyk, 2017a). The *exg1-1* mutant showed enhanced phloem reconnection at both 3 and 4 DAG and enhanced xylem reconnection compared with Col-0 plants at 7 DAG (Figure 1I and 1J; Supplemental Figure 1I and 1J). However, *EXG1-OE* significantly reduced phloem connectivity but had an insignificant effect on xylem connectivity compared with Col-0 (Figure 1I and 1J; Supplemental Figure 1I and 1J). We performed heterografting assays and found that *exg1-1* grafted as a scion on a Col-0 rootstock enhanced grafting, whereas overexpression of *EXG1* either in the scion or in the rootstock reduced grafting efficiency (Supplemental Figure 1K). We checked tissue attachment during grafting, since tissue attachment is needed for phloem and xylem connection (Melnyk et al., 2015; Melnyk and Meyerowitz, 2015). The *exg1-1* mutant improved tissue attachment at the graft junction, whereas *EXG1-OE* reduced tissue attachment compared with Col-0 (Supplemental Figure 1L). Since wounding typically produces wound-induced callus (Ikeuchi et al., 2017, 2020, 2022; Iwase et al., 2021), we tested this for *exg1-1* and found that it showed reduced wound-induced callus formation from petioles compared with Col-0; however, *EXG1-OE* exhibited little effect (Figure 1K and 1L). In hypocotyl callus, however, *exg1-1* showed an insignificant effect (Supplemental Figure 1M). The effects of *EXG1* appeared most prominent in regenerating tissues, since we could not detect any phenotypic differences between non-wounded Col-0 and *exg1-1* grown in pots (Supplemental Figure 1N). Next, we analyzed the predicted protein structure and subcellular localization of EXG1, and noticed that EXG1 is predicted to be a highly coiled extracellular protein and localized to the cell wall (Supplemental Figure 2A–2C). EXG1 also possesses a putative transmembrane domain and signal peptide motif at the N terminus, suggesting that it could be secreted (Supplemental Figure 2D and 2E; Supplemental Data 1) (Krogh et al., 2001; Jumper et al., 2021; Teufel et al., 2022; Varadi et al., 2022). To test the subcellular localization of EXG1, we generated an *EXG1pro:EXG1-mCherry* line and performed plasmolysis with 0.6 M sorbitol using *UBQpro:tdTomato* as a control. Post plasmolysis, we observed that, while tdTomato is localized with the plasma membrane, EXG1-mCherry is localized with the cell wall (Supplemental Figure 2F). Taken together, these results indicate that *EXG1* is cell-wall associated and activated by stress and wounding, and plays a role in suppressing graft formation and ectopic xylem formation.

EXG1 affects cambium development and nematode infection

To test the role of *EXG1* beyond wounding, we measured xylem morphology in *exg1* primary roots, since it affects ectopic xylem formation in leaves (Figure 1B–1D). We found that *exg1-1* had a greater number of metaxylem cell files compared with the wild-type Col-0 (Figure 2A). Cross sections 2 mm below the hypocotyl–root junction in 21-day-old seedlings showed reduced cambium and reduced xylem area in *exg1-1* compared with the wild-type Col-0 (Figure 2B–2D). We did not observe any changes in the number of xylem cells or the cambium-to-xylem area ratio between Col-0 and *exg1-1* (Figure 2E; Supplemental Figure 3A). However, when normalized for unit xylem area, *exg1-1* had more xylem cells per unit area than Col-0 (Figure 2F). Moreover, the total cross-sectional area was also lower in *exg1-1* compared with Col-0 (Supplemental Figure 3B). Next, we analyzed previously published datasets and found that *EXG1* was upregulated by multiple stress treatments including osmotic, salt, heat, drought, cold, UV-B, and nematode infection (Supplemental Figure 3C) (data from ePlant Browser, Bar Toronto) (Kilian et al., 2007; Fucile et al., 2011; Siddique et al., 2022; Waese et al., 2017) (Supplemental Figure 3D). We performed infection assays with the plant-parasitic cyst nematode, *H. schachtii*. Nematodes characteristically develop feeding sites near the vasculature and induce *de novo* phloem formation, assumed to symplastically connect the syncytial feeding structures to the vascular bundles for continuous supply of nutrients (Melnyk, 2017d). At the infection site, *exg1-1* had larger syncytium than the wild-type Col-0, whereas in *EXG1-OE*, the syncytium size was reduced compared with the wild-type Col-0 (Figure 2G and 2H). Twelve days post infection, the total number of male nematodes was significantly increased in *exg1-1* compared with *EXG1-OE* (Figure 2I). Female nematodes did not change in number but were reduced in size in *EXG1-OE*, while there were no changes in the combined total number of nematodes (Figure 2J and 2K; Supplemental Figure 3E). Taken together, these results suggest that, while *EXG1* promotes cambium formation, it suppresses xylem differentiation and inhibits syncytium development.

RLP44 mutants phenocopy *EXG1* mutants during development

Our finding that *exg1-1* displayed more xylem in VISUAL coupled to the observation of increased metaxylem cell files in primary roots prompted us to investigate the role of brassinosteroids, given that this hormone is known to promote xylem differentiation (Caño-Delgado et al., 2004; Ibañes et al., 2009; Furuya et al., 2021). We analyzed ectopic xylem formation using the VISUAL system for mutants in the brassinosteroid-signaling-related genes *BRASSINOSTEROID INSENSITIVE 1* (*BRI1*), *BRASSINOSTEROID INSENSITIVE 2* (*BIN2*), *BRI1-EMS SUPPRESSOR 1* (*BES1*), *BRASSINAZOLE RESISTANT 1* (*BZR1*), and *RLP44*. Brassinosteroid mutants have been previously implicated in affecting VISUAL (Kondo et al., 2014, 2015). We found that *bes1-2* and *bin2-1* showed reduced ectopic xylem formation, while *bes1-D* displayed increased ectopic xylem, compared with Col-0 (Figure 3A and 3B) (Kondo et al., 2015). However, we also found that *rlp44-3* had enhanced ectopic xylem formation, whereas *RLP44ox* showed a reduction in ectopic xylem formation compared with Col-0 (Figure 3A and 3B). The canonical brassinosteroid mutants, with the exception of *bri1-301*, showed little effect on metaxylem strand number in the primary root, but *rlp44-3* had extra metaxylem strands as previously reported (Figure 3C) (Holzwart et al., 2018) and similar to *exg1-1* (Figure 2A). We then treated *rlp44-3*, *bri1-301*, *bes1-2*, *bes1-D*, *bzr1-D*, and *bin2-1* with 10 nM epiBrassinolide (epiBL) or mock

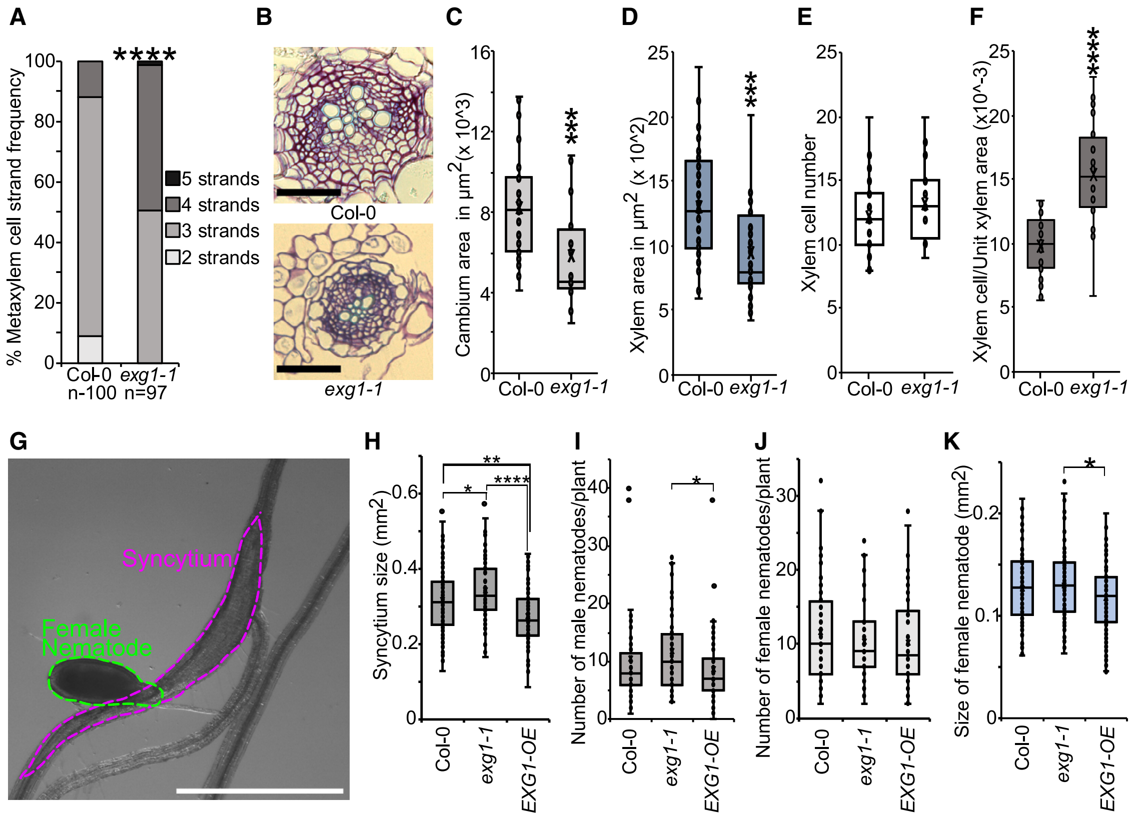

Figure 2. *EXG1* affects cambium development and nematode infection.
(A) Metaxylem strand number ($n \geq 97$).
(B) Cross sections 2 mm below 21-day-old shoot–root junction. Scale bars, 200 μm.
(C) Cambium area quantification. Dots represent individual samples.
(D) Xylem area quantification. Dots represent individual samples.
(E) Number of xylem cells. Dots represent individual samples.
(F) Xylem cell/unit xylem area quantification. Dots represent individual samples.
(G) Picture showing infection of Col-0 root by a female nematode (*Heterodera schachtii*). Green dotted line indicates female size, magenta dotted line indicates syncytium size. Scale bar, 1 mm.
(H) Syncytium size at 14 days post infection (dpi). Dots indicate individual samples.
(I) Number of male nematodes per plant at 12 dpi. Dots indicate individual samples.
(J) Number of female nematodes per plant. Dots indicate individual samples.
(K) Size of female nematodes at 14 dpi. Dots indicate individual samples.
Asterisks indicate statistical significance. For cross sections and nematode assay, significance was calculated by Wilcoxon's test: *$p < 0.05$, **$p < 0.01$, ***$p < 0.001$, and ****$p < 0.0001$. For metaxylem number, significance was calculated using Fisher's exact test with Benjamini–Hochberg adjustment: ****$p < 0.0001$.

conditions to observe if there are any developmental changes. epiBL rescued the metaxylem phenotype in all mutants. However, *bes1-2*, *bri1-301*, and *rlp44-3* were resistant to xylem identity changes compared with treated Col-0 (Supplemental Figure 4A and B). We also observed similar resistance to xylem identity changes in *exg1-1* roots when treated with epiBL (Supplemental Figure 4C). We then checked cross sections 2 mm below the hypocotyl–root junction in 21-day-old seedlings. We observed that in *rlp44-3*, both cambium area and xylem area were reduced compared with Col-0 (Figure 3D–3F), while *bes1-2* and *bri1-301* had no discernible reduction in cambium area compared with wild-type Col-0, but they showed reduced xylem area (Figure 3D–3F). Moreover, *bin2-1* showed a reduction in cambium and xylem area compared with Col-0 (Figure 3D–3F). The total cross-sectional area was reduced in *rlp44-3* and *bin2-1*, while no discernible difference was observed for the ratio of cambium to xylem area (Supplemental Figure 4D and 4E). Moreover, while all mutants had fewer xylem cells compared with Col-0, when normalized to unit xylem area, both *rlp44-3 and bin2-1* had more xylem cell per unit area (Supplemental Figure 4F). Overall, it appeared that only *rlp44-3* showed a complete overlap of phenotype with *exg1-1* during vascular development, suggesting that these two genes might share a common function.

RLP44 mutants phenocopy *EXG1* mutants during regeneration

To further understand the relationship between *RLP44* and *EXG1*, we analyzed their mutants during regeneration by performing grafting and callus formation assays. Expression of *RLP44* was initially repressed during graft formation but within 120 h increased compared with non-grafted controls (Figure 4A) (Melnyk et al.,

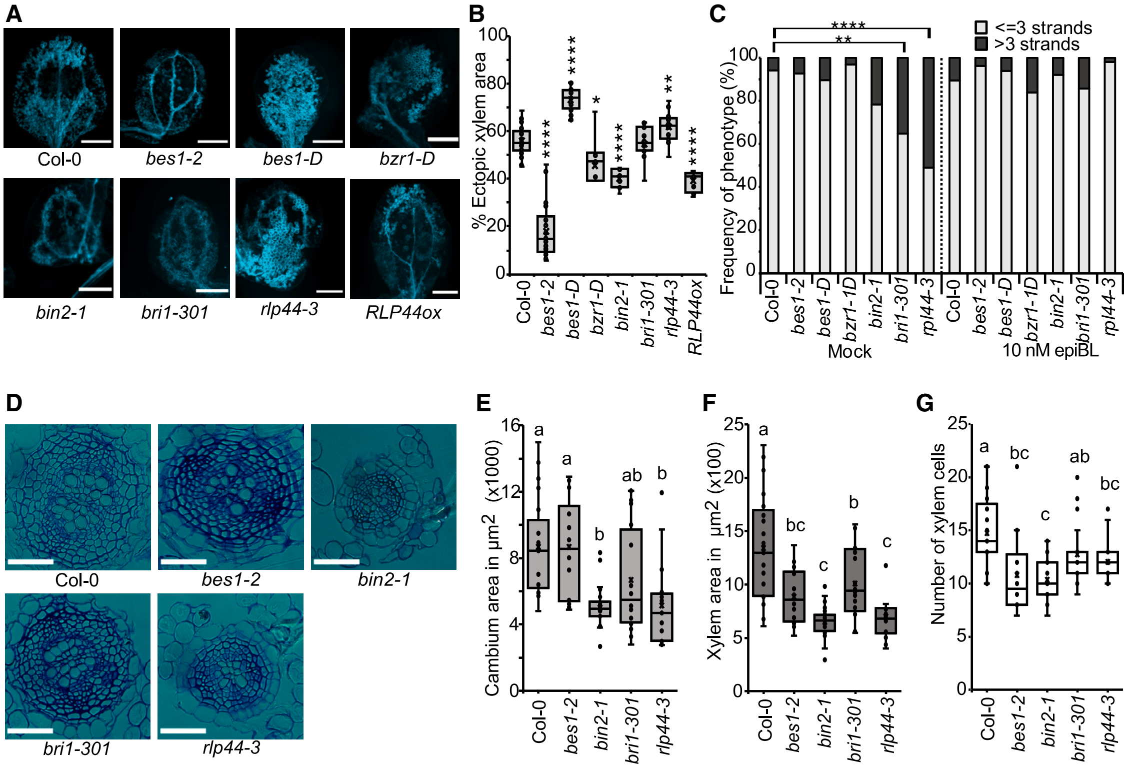

Figure 3. *RLP44* mutants behave like *EXG1* mutants in non-wounded conditions.

(A) Images showing bikinin-treated cotyledons of brassinosteroid-related mutants in VISUAL. Scale bars, 1 mm.

(B) Percentage of ectopic xylem area quantification. Dots represent samples.

(C) Metaxylem strand number under mock and epiBL treatments ($n \geq 32$).

(D) Cross sections 2 mm below the shoot–root junction. Scale bars, 50 μm.

(E) Cambium area comparison. Dots represent individual samples.

(F) Xylem area comparison. Dots represent individual samples.

(G) Xylem cell number comparison. Dots represent samples.

Asterisks indicate statistical significance compared to Col-0. For VISUAL, significance was calculate by Wilcoxon's test: *$p < 0.05$, **$p < 0.01$, and ****$p < 0.0001$. For metaxylem number, significance was calculated by Fisher's exact test with Benjamini–Hochberg adjustment: **$p < 0.01$ and ****$p < 0.0001$. For cross sections, significance was calculated by one-way ANOVA with Tukey's *post hoc* test. Lowercase letters indicate significant differences.

2018). Genes reported to be induced by brassinosteroids, such as *BR ENHANCED EXPRESSION 1* (*BEE1*), *BEE2*, *BEE3*, *PHYB ACTIVATION TAGGED SUPPRESSOR 1* (*BAS1*), *TOUCH4/XYLOGLUCAN ENDOTRANSGLUCOSYLASE/HYDROLASE 22* (*TCH4/XTH22*), *KIDARI/PRE6*, *SMALL AUXIN UPREGULATED-AC1* (*SAUR-AC1*), *INDOLE ACETIC ACID INDUCED 5* (*IAA5*), *IAA19*, and *VASCULAR RELATED NAC DOMAIN 6* (*VND6*) (Neff et al., 1999; Friedrichsen et al., 2002; Nakamura et al., 2004; Kubo et al., 2005; Vert et al., 2005; Liu et al., 2020), along with other core brassinosteroid pathway genes, showed some changes in gene expression during graft formation or petiole wounding, but there was no consistent pattern of up- or downregulation (Supplemental Figure 5A–5C) (Melnyk et al., 2018; Pan et al., 2019; Zhang et al., 2022). Grafting *bri1-301*, *bes1-2*, *bzr1-D*, and *bin2-1* reduced phloem and xylem reconnection, but exceptionally, *rlp44-3* showed increased rates of phloem connectivity compared with Col-0 at both 3 and 4 DAG and had a slight but non-significant increase in xylem connectivity (Figure 4B and 4C; Supplemental Figure 5D). We obtained a *35Spro:RLP44-RFP* (*RLP44ox*) line (Wolf et al., 2014) and found that it behaved opposite to *rlp44-3*, showing reduced phloem and xylem connectivity (Figure 4B and 4C). In addition, *rlp44-3* exhibited improved attachment of the grafts (Figure 4D). Heterografting assays revealed that mutations in *RLP44* in the rootstock reduced phloem reconnection, whereas its mutations in the scion non-significantly improved grafting (Supplemental Figure 5E and 5F). As a second test, we analyzed callus formation levels in wounded petiole explants and observed that only *bes1-D* and *rlp44-3* showed significantly reduced petiole callus formation compared with Col-0 (Figure 4E and 4F). Taken together, these results show that, while core brassinosteroid signaling promotes vascular regeneration and callus formation, mutations in *RLP44* exihbit regeneration phenotypes similar to those of mutations in *EXG1*, and both genes appear to repress vascular connectivity but promote petiole callus formation. Moreover, our results also suggest that, while *RLP44* mutants show similarities to *EXG1* mutants, these phenotypes are unlike to those of canonical brassinosteroid signaling mutants, and these genes potentially act through a different pathway.

EXG1 and *RLP44* affect a common set of stress-responsive and cell-wall-related genes

Our previous data suggest that *EXG1* and *RLP44* might regulate similar pathways. Thus, we compare their expression patterns.

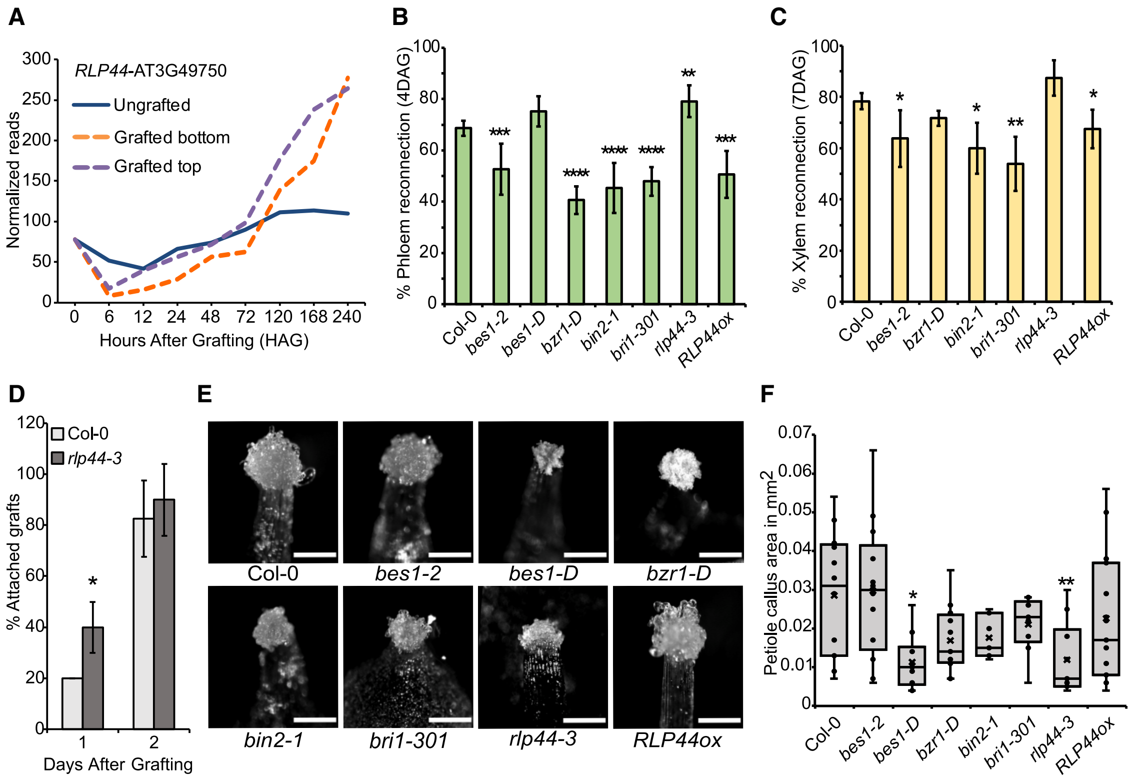

Figure 4. *RLP44* mutants behave like *EXG1* mutants during regeneration.

(A) *RLP44* expression during graft formation (Melnyk et al., 2018).

(B and C) Reconnection percentage of phloem (4 DAG) and xylem (7 DAG) in homografted brassinosteroid-related mutants. Mean ± SD of three to seven experiments is shown.

(D) Attachment rate. The mean ± SD of three experiments is shown.

(E) Images showing callus formation in petiole explants. Scale bars, 200 μm.

(F) Petiole callus area quantification. Dots indicate samples.

Asterisks indicate significant difference compared to Col-0. For grafting assays, significance was calculated by pairwise *t*-tests with Benjamini–Hochberg adjustment: *$p < 0.05$, **$p < 0.01$, ***$p < 0.001$, and *****$p < 0.0001$. For attachment rates, significance was calculated by Student's *t*-test: *$p < 0.05$. For petiole callus assays, significance was calculated using Wilcoxon's test: *$p < 0.05$ and **$p < 0.01$.

EXG1 and *RLP44* translational reporters showed different expression patterns: *EXG1* was primarily epidermal in root tips, while *RLP44* was in the inner vascular tissues and lateral root cap (Figure 5A). However, in hypocotyls, *EXG1* was expressed in multiple cell files, and we could observe its vascular expression similar to *RLP44* (Figure 5B). We also found that *EXG1* transcripts were highly and rapidly induced upon cutting or wounding, whereas *RLP44* transcripts were delayed but increased as time progressed (Pan et al., 2019; Matosevich et al., 2020) (Supplemental Figure 6A and 6B). We performed grafting with the translational reporters and found that after cutting, *EXG1* and *RLP44* reporters were activated in the vascular region (Figure 5B). A similar co-expression pattern of *EXG1* and *RLP44* was also observed in the vasculature in cut petioles (Supplemental Figure 6C). To understand whether *exg1-1* affects any cambium-related genes, we performed qPCR assays with markers for cambium (*WOX4* and *ATHB8*) and xylem (*VND6*) but found no discernible difference in transcript levels when comparing mutants with Col-0 (Supplemental Figure 6D–6F).

We next performed genome-wide transcriptomic analyses on *exg1-1* and *rlp44-3* seedlings to identify differentially expressed genes (DEGs). We found 977 DEGs in *exg1-1* compared with the wild-type Col-0, with 369 genes downregulated and 608 genes upregulated (Supplemental Data 2). In *rlp44-3*, we identified 171 DEGs, including 129 genes upregulated and 42 genes downregulated compared with the wild-type Col-0 (Supplemental Data 3). We performed a Gene Ontology (GO) analysis on *exg1-1*-regulated transcripts and observed a large and significant enrichment for cellular components associated with the cell wall (Supplemental Data 4). Cell-wall-related genes such as *XYLOGLUCAN ENDOTRANSGLUCOSYLASE/HYDROLASE 19* (*XTH19*), *XTH20*, *XTH31*, and *TRICHOME BIREFRINGENCE-LIKE 15* (*TBL15*) were downregulated, while *XTH4*, *XTH6*, *XTH16*, *XTH22*, *XTH24*, and *XTH27* were upregulated. Genes enriched for cell-wall loosening, including *EXPANSIN B3* (*EXPB3*), *EXPA3*, *EXPA4*, *EXPA5*, *EXPA8*, and *EXPA15*, were also downregulated (Supplemental Data 2). Genes related to stress response, such as *RESPONSE TO DESICCATION 29A* (*RD29A*), *KIN1*, *COR6.6*, *COLD RELATED 15B* (*COR15B*), and *ETHYLENE RESPONSE FACTOR 104* (*ERF104*), were also differentially expressed. We then compared the gene expression profiles of *exg1-1* and *rlp44-3*, and observed a significant overlap between their profiles (Figure 5C). We selected a subset of genes based on those most highly expressed and analyzed their transcription dynamics during graft formation based on an existing dataset (Melnyk et al., 2018). We found that the overlapping upregulated genes in

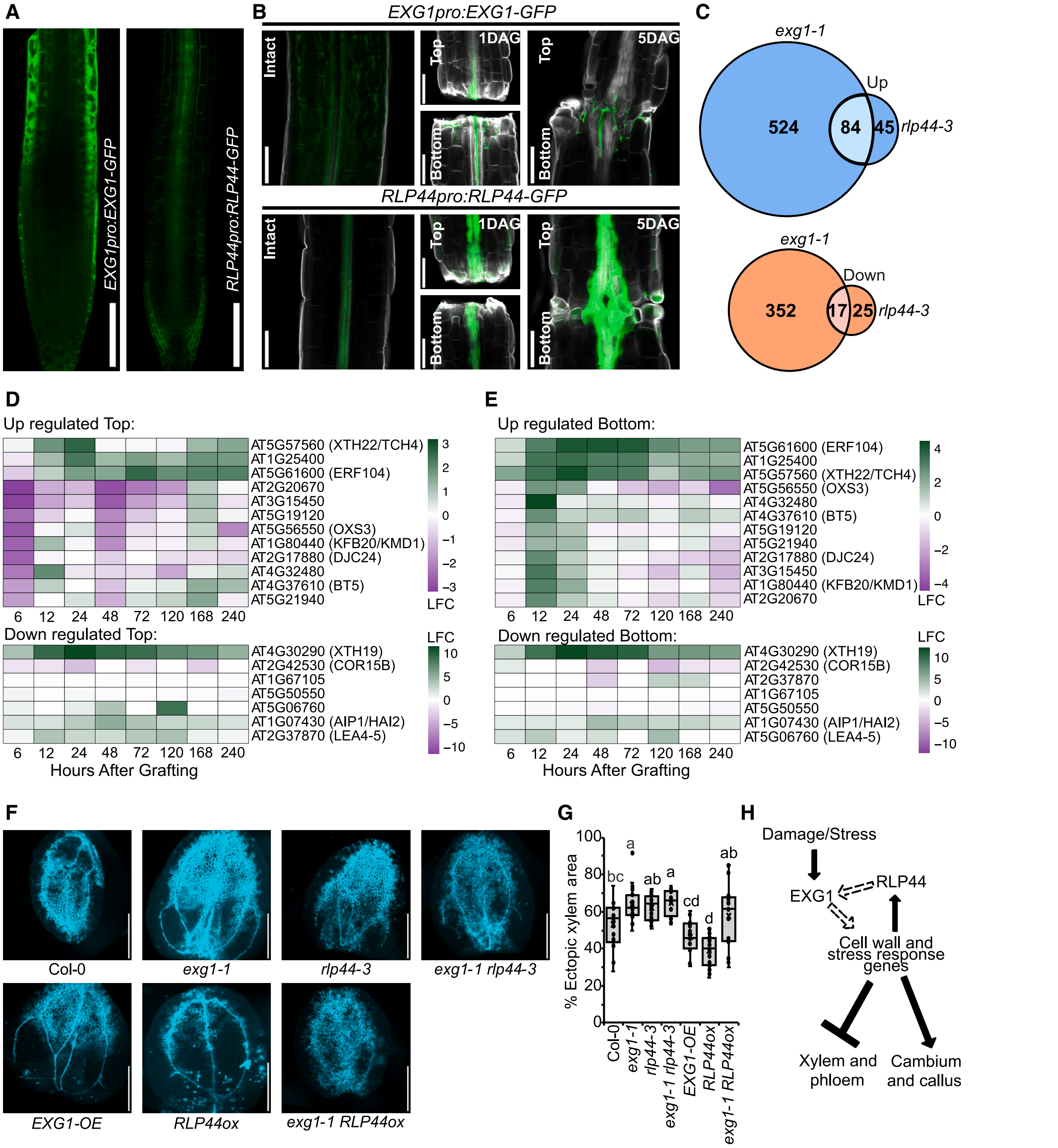

Figure 5. *EXG1* and *RLP44* share common target genes that modify regeneration potential.
(A) *EXG1pro:EXG1-GFP* and *RLP44pro:RLP44-GFP* fusion protein fluorescence in root tips. Scale bars represent 100 μm.
(B) *EXG1pro:EXG1-GFP* and *RLP44pro:RLP44-GFP* fusion protein fluorescence during grafting at 1 and 5 DAG compared to intact. Cell walls were stained by Calcofluor white (gray). Scale bars represent 100 μm.
(C) Venn diagram representing overlap between differentially expressed genes (DEGs) in *exg1-1* and *rlp44-3*. $p = 1.114E-28$ for overlapping downregulated genes and 1.511E−35 for overlapping upregulated genes by hypergeometric test.
(D) Heatmap of common up- and downregulated DEGs between *exg1-1* and *rlp44-3* in WT Col-0 grafted top during graft formation compared to ungrafted Col-0 (Melnyk et al., 2018).
(E) Heatmap of common up- and downregulated DEGs between *exg1-1* and *rlp44-3* in WT Col-0 grafted bottom during graft formation compared to ungrafted Col-0 (Melnyk et al., 2018).
(F) Images of samples in VISUAL.

(legend continued on next page)

the genome-wide transcriptomic analyses were also those highly upregulated in the grafted bottom, whereas overlapping downregulated genes showed no clear trend (Figure 5D and 5E). These results demonstrate that *EXG1* and *RLP44* affect a common set of genes and provide targets in the rootstock that might explain changes in grafting success. We selected *EXORDIUM LIKE 3* (*EXL3*), which was one of the 22 genes shortlisted (Figure 1A) and a common upregulated gene in *exg1-1* and *rlp44-3* (Supplemental Figure 6G; Supplemental Data 2 and 3). An *exl3* mutant showed reduced graft phloem reconnection and reduced VISUAL ectopic xylem formation (Supplemental Figure 6H–6J), consistent with this gene being important for vascular formation and a possible candidate for how *exg1* and *rlp44* mutations enhanced grafting.

To understand the biological relationship between *EXG1* and *RLP44*, we generated various double-mutant combinations (Supplemental Figure 6K). We analyzed ectopic xylem formation with VISUAL and observed that *exg1-1 rlp44-3* showed similar enhanced ectopic xylem levels compared with *exg1-1* and *rlp44-3* single mutants (Figure 5F and 5G). The *exg1-1 RLP44ox* plants had lower ectopic xylem levels more similar to those of *exg1-1* (Figure 5F and 5G). A similar trend was also observed with the metaxylem cell file numbers in the roots of *exg1-1 RLP44ox* plants (Supplemental Figure 6L). These data suggest that *EXG1* and *RLP44* act in the same genetic pathway and that *EXG1* function is required for the *RLP44ox* phenotype.

DISCUSSION

In this study, we identified *EXG1* as a stress-responsive gene that represses vascular differentiation. Upon wounding, induction of *EXG1* was rapid, occurring between 10 min and 6 h after cutting (Figures 1 and 6), suggesting its regulation by hormones, reactive oxygen species, cell-wall modifications, turgor pressure, or other rapid responses (Hoermayer et al., 2020; Bellandi et al., 2022; Zhang et al., 2022). *EXG1* upregulation occurred with various biotic and abiotic stressors that cause tissue damage or tissue invasion, demonstrating a common plant response. Unlike short-acting defense responses that occur during plant grafting (Melnyk et al., 2018), *EXG1* induction lasted for several days, suggesting it had developmental roles during regeneration. *exg1* mutants showed reduced callus formation yet enhanced phloem connectivity, xylem connectivity, and tissue attachment during wounding or grafting. To our knowledge, *exg1* is the first identified recessive mutation that enhances grafting success. These phenotypes were surprising, given the rapid and strong induction of *EXG1* by grafting, suggesting that induction of a negative regulator is of benefit. Moreover, *exg1* increased graft attachment rates but reduced cambium levels (Figures 1 and 2). Given the importance of cambium during grafting (Melnyk, 2017c; Melnyk et al., 2018), our results seem paradoxical but could be explained by an enhancement of attachment or earlier vascular differentiation in *exg1* despite limited cambium formation. We did not observe any vascular- or cambium-related genes differentially expressed in *exg1* (Supplemental Figure 6). In contrast, we found genes differentially expressed in *exg1* that were related to cell-wall dynamics and modifications (Supplemental Data 2 and 4). We propose that *EXG1* contributes to cambial divisions but represses vascular differentiation by affecting cell-wall-related genes. Such cell-wall changes could explain the enhanced tissue attachment, which is often pectin related during grafting (Feng et al., 2024), and changes in cambium cell size (Figures 1 and 2) in its mutants. Modifying cell-wall-related genes, including a pectin lyase-like gene, is known to affect phloem, xylem, and cambium formation (Bush et al., 2022; Kalmbach et al., 2023), consistent with a link between cell walls and vascular development. *EXG1* also plays important developmental roles under non-stress conditions and might promote cell division at the expense of cellular differentiation during vascular development.

The molecular and biochemical function of EXG1 remains elusive, but *in silico* analyses have found two Domains of Unknown Function 642 (DUF642) in the EXG1 protein (Vázquez-Lobo et al., 2012). DUF642 is a highly conserved protein family in spermatophytes involved in cell-wall modification, cell-wall maintenance (Salazar-Iribe et al., 2016; Cruz-Valderrama et al., 2019), and, in *Amaranthus*, abiotic stress response (Palmeros-Suárez et al., 2017). EXG1 has also been found in cell-wall proteomes and interacts with cellulose and hemicellulose *in vitro* (Borner et al., 2003; Ndimba et al., 2003; Vázquez-Lobo et al., 2012). Our own analyses suggest that the EXG1 protein is localized in the cell wall or extracellular space (Supplemental Figure 2A–2F; Supplemental Data 4) (Krogh et al., 2001; Jumper et al., 2021; Teufel et al., 2022; Varadi et al., 2022). Analyses of DEGs in *exg1* revealed that many genes related to cell-wall organization, biogenesis, and cell-wall loosening. *XTH4*, which affects xylem cell expansion and secondary cell-wall development (Kushwah et al., 2020), were upregulated when *EXG1* function was lost. Multiple genes encoding expansins such as *EXPA4*, *EXPA8*, *EXPA16*, and *EXPB3* were also downregulated in *exg1*. We thus speculate that *EXG1* appears to be closely linked to cell-wall function and cell-wall signaling. Previous analyses found that *EXG1* mRNA is highly expressed in developing seeds. Thus, and it is possible that *EXG1* is relevant to some aspects of seed germination such as pectin-related mucilage formation (Vázquez-Lobo et al., 2012; Garza-Caligaris et al., 2012). Further work is needed to understand EXG1's precise molecular function and its function in the cell wall, including interactions with pectin and cellulose.

Although we obtained little evidence for a consistent brassinosteroid response at the graft junction, many brassinosteroid genes were needed for efficient grafting success (Figure 4). *RLP44* was exceptional since it repressed grafting success. Previously, this gene has been implicated in promoting cambium and repressing xylem formation (Wolf et al., 2014; Holzwart et al., 2018). In this study, we verified and extended these observations to demonstrate that *RLP44* was activated by wounding and

(G) Percentage of ectopic xylem area quantification. Dots represent individual samples. Significance was calculated by one-way ANOVA with Tukey's *post hoc* test. Lowercase letters indicate significant differences.

(H) A proposed model depicting the possible action mechanism of *EXG1* and *RLP44*. Damage or stress activates *EXG1* to potentially cause changes in cell walls. Such changes are perceived by RLP44, which modifies EXG1 function further. The interplay between EXG1 and RLP44 causes downstream phenotypic and developmental changes.

suppressed vascular regeneration and graft formation (Figures 3 and 4). Although *RLP44* and *EXG1* mutants were phenotypically indistinguishable in our assays, the cellular localization of *RLP44* was primarily in the vascular cylinder, whereas *EXG1* was primarily in the epidermis and cortex in root tips and more broadly expressed in hypocotyls (Figure 5A and 5B). During grafting or wound healing, *EXG1* was activated earlier than *RLP44*, and both proteins were induced in similar tissues (Figure 5B; Supplemental Figure 6C). The differential activation of *RLP44* and its non-overlapping expression pattern with *EXG1* suggest that these two proteins may have independent cell-specific roles or, potentially, proteins or RNAs are cell-to-cell mobile so as to function in the same cell. Cell-wall-derived signals are proposed to act non-autonomously to regulate vascular development (Bush et al., 2022), and *EXG1* may be related to such a signal that promotes cell proliferation.

The transcriptomic overlap between *exg1* and *rlp44* identified numerous genes regulated in common, including *EXL3*, *XTH19*, *COR15B*, *HIGHLY ABA-INDUCED PP2C GENE 2* (*HAI2*), *XTH22*, *ERF104*, and *AT1G25400*, many of which have been implicated in cell-wall changes (Cosgrove, 2000, 2016; Miedes et al., 2013). Such overlapping genes may present factors that distinguish cell division from differentiation and deserve further investigation. Our epistasis analysis found that single and double mutants of *EXG1* and *RLP44* looked phenotypically the same, suggesting these genes may act in the same pathway (Figure 5F and 5G; Supplemental Figure 6K). A combined line between *exg1* and *RLP44ox* also supported a role for *EXG1* acting downstream of, or together with, *RLP44*, although RNA levels of the *RLP44* transgene were slightly reduced in the *exg1* background (Supplemental Figure 6M and 6N). Such activation of two negative regulators during graft formation could be important to balance or modify the speed of cambial proliferation and cellular differentiation. In summary, we propose a model in which *EXG1* responds to stress and, through interactions with *RLP44*, mediates cell-wall signaling to repress vascular differentiation and promote cambial proliferation (Figure 5H). Such a framework could help modify grafting success and the regenerative abilities of plants.

METHODS

Plant material and growth conditions

A. thaliana accession Col-0 was used as the wild-type control in this study, and all mutants used are in the Col-0 background unless mentioned otherwise. A list of mutants used in this study is described in Supplemental Table 1. Primers used for checking homozygosity or for transcript quantification are described in Supplemental Tables 2 and 3. Seeds were surface sterilized with 75% (v/v) ethanol for 20 min and then 99.5% (v/v) ethanol for 5 min and then dried in a sterile hood. Sterilized seeds were then placed on half-strength Murashige and Skoog (MS) (Murashige and Skoog, 1962) medium with 1.2% plant agar unless mentioned otherwise. Seeds stratified for 48–72 h in 4°C were moved to the growth chamber under short-day conditions (8 h light/16 h dark, ~110 mmol m^{-2} s^{-1}, 20°C, Conviron A1000 chamber) or long-day conditions (16 h light/8 h dark, 120 mmol m^{-2} s^{-1}, 22°C day temperature, and 20°C night temperature) unless mentioned otherwise. Plates were kept vertically for vertical plant growth.

Plasmid construction and transgenic line generation

To generate *EXG1pro:GFP* and *EXG1pro:EXG1-GFP*, a 2112 bp promoter region as described in Salazar-Iribe et al. (2016) and the *EXG1* coding sequence without a stop codon were cloned into the promoter module (A–B overhang) and CDS module (C–D overhang), respectively, in the GreenGate cloning system (Lampropoulos et al., 2013). Following the cloning protocol, all necessary modules, including the GFP coding sequence module (C–D overhang) for *EXG1pro:GFP*, the GFP linker sequence (D–E overhang), and the mCherry linker sequence (D–E overhang) module were used in the final cloning reaction to create *EXG1pro:GFP*, *EXG1pro:EXG1-GFP*, and *EVP1pro:EXG1-mCherry*, respectively. The module for selection was obtained from pHDE-35S-Cas9-mCherry-UBQ, which was a gift from Yunde Zhao (Addgene plasmid no. 78932; http://n2t.net/addgene:78932; RRID: Addgene_78932) (Gao et al., 2016). Transgenic lines were generated using the floral dip method (Clough and Bent, 1998). All primers used for cloning are listed in Supplemental Table 4.

Ectopic xylem formation in cotyledons

Ectopic xylem formation assays were performed according to a previously published method VISUAL (Kondo et al., 2015, 2016), with one minor change in the induction medium with the addition of BF-170 (Nurani et al., 2020), a lignin-binding secondary cell-wall indicator for easier imaging of xylem. In brief, *Arabidopsis* seeds were grown for 6 days under 24 h light conditions. The cotyledons were then excised and transferred to induction medium. At the end of the 4-day induction period, cotyledons were fixed overnight in a solution of acetic acid and 99.5% ethanol (1:3, v/v). Samples were then placed in a chloral hydrate solution and mounted on slides with chloral hydrate for visualization of autofluorescence (UV filter) with a Leica M205 FA stereo fluorescence microscope. The area of ectopic xylem was calculated from autofluorescence levels using Fiji and normalized to the total cotyledon area. Cotyledon veins were excluded from the quantification.

Plant micrografting and attachment

Seven-day-old short-day-grown seedlings were used for micrografting (Melnyk, 2017b). In brief, for attachment assays, grafted plants were picked up with forceps at the root–hypocotyl junction and placed back down at 1 and 2 DAG. If the scion remained attached during the entire movement, the plant was scored as positive for attachment. Percentage attached grafts was calculated as a function of the number of attached grafts to the total grafted plants. For the phloem reconnection assay, the cotyledon was damaged with forceps, and CFDA was placed on the wound site. Phloem reconnection was scored successful if the fluorescent signal appeared in the root after 1 h at tested time points. Percentage phloem reconnection was calculated as a function of the number of plants with fluorescent roots versus number of plants grafted. New plants were used for each time point. For the xylem reconnection assay, the root was cut 1–2 cm below the hypocotyl and then CFDA was dropped on the wound site. After 20 min, xylem reconnection was scored successful if the fluorescent signal was found in the cotyledon at tested time points. Percentage xylem reconnection was calculated as a function of the number of plants with fluorescent cotyledons versus number of plants grafted. New plants were used for each time point. For imaging reporters during grafting, tissues were collected 1 and 5 DAG, fixed with 4% paraformaldehyde in PBS for 10 min, and cleared with ClearSee solution following a modified protocol from Ursache et al. (2018)

Callus regeneration and wounding assays

Callus induction in petiole explants was performed using a previously published method with some changes (Iwase et al., 2017). Cotyledons with petioles were excised from 10-day-old, long-day-grown seedlings. They were then placed on full-strength MS medium plates supplemented with 1% sucrose and 0.6% Gelrite under long-day conditions. Callus induction in hypocotyl explants was performed using a previously published method with some changes (Iwase et al., 2011b). The seeds were grown in the dark to generate etiolation on MS medium supplemented with 0.05% MES, 0.5% sucrose, and 0.8% Gelrite. Seven days of dark growth was followed with a cut that was performed at approximately 7 mm above

the hypocotyl–root junction to induce callus. After 8 days of induction, sample tissues from both petiole explants and hypocotyl explants were imaged with a Leica M205 FA stereo fluorescence microscope. Projected callus area in the image was measured using the freehand tool in Fiji. For imaging reporters during callus regeneration, tissues were collected 3 and 4 days post infection (dpi), fixed with 4% paraformaldehyde in PBS for 10 min, and cleared with ClearSee solution following a modified protocol from Ursache et al. (2018).

Histological sections

To avoid lateral roots, 21-day-old long-day-grown seedling samples were cut 2 cm below the shoot tip and were collected and vacuum infiltrated using a fixation solution (1% glutaraldehyde, 4% formaldehyde, and 0.05 M sodium phosphate). After keeping in the fixation solution for at least overnight and subsequent ethanol dehydration, the samples were oriented with shoot pointing to the top in a mold, with the leaves removed. The samples were then infiltrated and embedded with Leica Historesin. Cross sections (2.5 μm thick) were cut 2 mm below the shoot–root junction with a Leica microtome, followed by staining with toluidine blue and imaging with a Zeiss Axioscope A1 microscope.

Plasmolysis

Plants were stained with 0.1% Calcofluor white dissolved in water or 0.1% Calcofluor white dissolved in 0.6 M sorbitol for 15 min and then washed with water or 0.6 M sorbitol, respectively. Images were taken with a Zeiss LSM780 inverted Axio Observer. For Calcofluor white, 405 nm excitation and 410–451 nm emission were used; for reporter lines expressing mCherry, 561 nm excitation and 598–696 nm emission; and for tdTomato, 561 nm excitation and 576–691 nm emission. Images were taken with a 63× water objective with 2× digital magnification.

Confocal microscopy

For confocal microscopy, roots were mounted in 10 μM propidium iodide (PI) solution between two coverslips and imaged immediately. Confocal micrographs were captured using a Zeiss LSM780 inverted Axio Observer with GaAsP detectors for *EXG1pro:GFP* and Zeiss LSM800 for *EXG1pro:EXG1-GFP* and *RLP44pro:RLP44-GFP*. For reporter lines expressing GFP and stained with PI, 488 nm excitation and 500–553 nm emission were used for both GFP and PI signals. For analysis of fluorescence during grafting, grafted plants were mounted on water between two coverslips and analyzed 24 h after grafting.

Root xylem architecture

Sterilized and stratified seeds were placed on 25-mm-pore Sefar Nitex 03-25/19 mesh (Ramachandran et al., 2018) on a half-strength MS plate supplemented with 1.2% plant agar and then grown vertically in long-day conditions for 3 days. After 3 days the plants were transferred to half-strength MS, 1.2% plant agar plates supplemented with DMSO, or 10 nM epiBL and kept vertically in long-day conditions for an additional 3 days. Roots were mounted on chloral hydrate solution and imaged at 40× with a Zeiss Axioscope A1 with differential interference contrast to analyze xylem morphology.

RNA isolation and quantitative real-time PCR

Total RNA was isolated using a Roti-Prep RNA MINI Kit. RNA samples were quantified using a NanoDrop ND-1000 spectrometer (Thermo Fisher Scientific). cDNA was prepared using 500 ng of total RNA using a Maxima First Strand cDNA Synthesis Kit containing oligo(dT) and random hexamer primers. The cDNA was diluted 1:9 with nuclease-free water. The iCycler iQ real-time PCR detection system with 10 μl reaction volumes (5 μl of 2× Maxima SYBR Green qPCR/ROX Master Mix, 1.2 μM forward and reverse primers, and 2.5 μl of diluted cDNA) was used to perform the qPCR. The program used for real time-qPCR was as follows: initial denaturation for 10 min at 95°C followed by 40 cycles of 95°C for 30 s and 60°C for 30 s. This was followed by a melt-curve analysis. Relative expression levels of selected genes were calculated using the $2^{-\Delta\Delta CT}$ method (Livak and Schmittgen, 2001). For analysis of transcript levels in *exg1-1* and *exg1-2*, *UBC9*, *TIP41-like*, and *PP2A* were used as loading references (Czechowski et al., 2005).To analyze *EXG1* transcript levels during VISUAL, *APT1* was used as a loading reference (Gutierrez et al., 2008). Three biological replicates were prepared for each genotype.

Preparation, sequencing and analysis of transcriptomic library

For RNA sequencing library preparation, 200 ng of total RNA extracted from 7-day old, short-day-grown seedlings was treated using a Poly(A) mRNA Magnetic Isolation Module kit. The library was prepared with the resulting mRNA using a NEBNext Ultra II Directional RNA Library Prep Kit for Illumina and NEBNext Multiplex Oligos for Illumina. Libraries were sequenced at Novogene on a NovaSeq 6000 in 150 bp paired-end mode. For RNA sequencing analyses, the raw data were cleaned using fastp to remove the low-quality reads (Chen et al., 2018). Hisat2 was used to map the cleaned reads to the *Arabidopsis* reference TAIR10 (Kim et al., 2015). Counts of reads were determined using HTseq-count (Anders et al., 2015). DEGs were defined using the DESeq2 R package. Genes with an adjusted $p < 0.05$ were considered to have statistically significant expression differences between samples, with wild-type Col-0 as the reference. The list of DEGs between *exg1-1* and Col-0 is provided in Supplemental Data 2. GO term enrichment analysis for *exg1-1* DEGs was performed using the GO term enrichment tool on TAIR relying on PANTHER (Ashburner et al., 2000; Mi and Thomas, 2009; Mi et al., 2013, 2019, 2021; Gene Ontology Consortium et al., 2021) and is provided in Supplemental Data 4. The list of DEGs between *rlp44-3* and Col-0 is provided in Supplemental Data 3. Heatmaps show the expression of common up- and downregulated genes in *exg1-1* and *rlp44-3* in wild-type Col-0 grafted top and grafted bottom during graft formation compared to ungrafted Col-0 by calculating log2(grafted/ungrafted) (Melnyk et al., 2018). A threshold of log2FC $\geq$ 1.5 or ≤ -1.5 for up- or downregulation in either or both *exg1-1* and *rlp44-3* was selected.

Nematode infection assays

The nematode infection was performed following the protocol mentioned in previous reports (Anjam et al., 2020). Briefly, 12-day-old *Arabidopsis* plants grown on modified Knop medium were infected with approximately 100 freshly hatched surface-sterilized second-stage juvenile *H. schachtii*. Twelve days post infection, developed male and female nematodes were counted using a Leica MZ16 stereo zoom microscope. At 14 dpi, syncytia and females were imaged using a Leica MZ16 stereo zoom microscope mounted with a Leica MC190HD camera. The area of the corresponding images was measured using ImageJ.

Statistical analyses

All statistical analyses were performed using R Studio with R version 4.2.0. Student's *t*-test with two-tailed distribution or pairwise *t*-test with Benjamini–Hochberg adjustment was used to compare two groups in the case of normal distribution; otherwise, Wilcoxon's signed rank test was used. For categorical values, Fisher's exact test with Benjamini–Hochberg correction was used. For comparison between multiple groups, one-way ANOVA followed by a *post hoc* Tukey HSD test was performed. A $p < 0.05$ was considered statistically significant.

DATA AND CODE AVAILABILITY

All data are available from the corresponding author upon request. mRNA sequencing data from this study have been deposited in the Gene Expression Omnibus database https://www.ncbi.nlm.nih.gov/geo under accession GEO: GSE224565. Other sequence data from this article can be found in the EMBL/GenBank data libraries under the following accession numbers: *EXG1*, AT3G08030; *CIPK5*, AT5G10930; *LTPG5*, AT3G22600; *SVB5*, AT4G24130; *RLP44*, AT3G49750; *EXL3*, AT5G51550; *BRI1*, AT4G39400; *BES1*, AT1G19350; *BZR1*, AT1G75080; and *BIN2*, AT4G18710.

FUNDING

S.M. and C.W.M. were supported by a Vetenskapsrådet grant (2017-05122). A.Z. and C.W.M. were supported by a Wallenberg Academy Fellowship (2016-0274). F.A., C.M. and C.W.M. were supported by a European Research Council starting grant (GRASP-805094). M.S.A. and P.M. were supported by a Vetenskapsrådet grant (2019-05634) and an MSCA Postdoctoral Fellowship (101066035-PREENER).

ACKNOWLEDGMENTS

We thank the Nottingham Arabidopsis Seed Center (NASC), Sebastian Wolf (University of Tübingen, Germany), Yuki Kondo (Kobe University, Japan), and RIKEN BRC for materials. We would also like to thank Igor Sabljic (Swedish University of Agricultural Sciences) for help with protein prediction software and Abdul Kareem V.K. (Swedish University of Agricultural Sciences) for confocal microscopy assistance. No conflict of interest is declared.

AUTHOR CONTRIBUTIONS

S.M. and C.W.M. designed the study. S.M. and F.A. conducted the experiments, analyzed the data, and designed the figures. A.Z. performed the transcriptomic analyses. C.M. performed the histological sections. C.M. and S.M. analyzed the histological data. M.S.A. and P.M. performed and analyzed nematode infection data. S.M. and C.W.M. wrote the manuscript. All authors approved the final manuscript. Funding acquisition was by M.S.A., P.M., and C.W.M.

SUPPLEMENTAL INFORMATION

Supplemental information is available at *Plant Communications Online*.

Received: October 16, 2024
Revised: December 19, 2024
Accepted: January 11, 2025
Published: January 16, 2025

REFERENCES

Anders, S., Pyl, P.T., and Huber, W. (2015). HTSeq–a Python framework to work with high-throughput sequencing data. Bioinformatics **31**:166–169.

Anjam, M.S., Shah, S.J., Matera, C., Różańska, E., Sobczak, M., Siddique, S., and Grundler, F.M.W. (2020). Host factors influence the sex of nematodes parasitizing roots of *Arabidopsis thaliana*. Plant Cell Environ. **43**:1160–1174.

Ashburner, M., Ball, C.A., Blake, J.A., Botstein, D., Butler, H., Cherry, J.M., Davis, A.P., Dolinski, K., Dwight, S.S., Eppig, J.T., et al. (2000). Gene ontology: tool for the unification of biology. Nat. Genet. **25**:25–29.

Baima, S., Possenti, M., Matteucci, A., Wisman, E., Altamura, M.M., Ruberti, I., and Morelli, G. (2001). The Arabidopsis ATHB-8 HD-Zip Protein Acts as a Differentiation-Promoting Transcription Factor of the Vascular Meristems. Plant Physiol. **126**:643–655.

Barcala, M., García, A., Cabrera, J., Casson, S., Lindsey, K., Favery, B., García-Casado, G., Solano, R., Fenoll, C., and Escobar, C. (2010). Early transcriptomic events in microdissected Arabidopsis nematode-induced giant cells. Plant J. **61**:698–712.

Bellandi, A., Papp, D., Breakspear, A., Joyce, J., Johnston, M.G., de Keijzer, J., Raven, E.C., Ohtsu, M., Vincent, T.R., Miller, A.J., et al. (2022). Diffusion and bulk flow of amino acids mediate calcium waves in plants. Sci. Adv. **8**:eabo6693.

Borner, G.H.H., Lilley, K.S., Stevens, T.J., and Dupree, P. (2003). Identification of Glycosylphosphatidylinositol-Anchored Proteins in Arabidopsis. A Proteomic and Genomic Analysis. Plant Physiol. **132**:568–577.

Bush, M., Sethi, V., and Sablowski, R. (2022). A phloem-expressed pectate lyase-LIKE gene promotes cambium and xylem development. Front. Plant Sci. **13**:888201.

Canher, B., Heyman, J., Savina, M., Devendran, A., Eekhout, T., Vercauteren, I., Prinsen, E., Matosevich, R., Xu, J., Mironova, V., and De Veylder, L. (2020). Rocks in the auxin stream: Wound-induced auxin accumulation and *ERF115* expression synergistically drive stem cell regeneration. Proc. Natl. Acad. Sci. USA **117**:16667–16677.

Caño-Delgado, A., Yin, Y., Yu, C., Vafeados, D., Mora-García, S., Cheng, J.-C., Nam, K.H., Li, J., and Chory, J. (2004). BRL1 and BRL3 are novel brassinosteroid receptors that function in vascular differentiation in *Arabidopsis*. Development **131**:5341–5351.

Chen, S., Zhou, Y., Chen, Y., and Gu, J. (2018). fastp: an ultra-fast all-in-one FASTQ preprocessor. Bioinformatics **34**:i884–i890.

Clough, S.J., and Bent, A.F. (1998). Floral dip: a simplified method for Agrobacterium-mediated transformation of Arabidopsis thaliana. Plant J. **16**:735–743.

Cosgrove, D.J. (2000). Loosening of plant cell walls by expansins. Nature **407**:321–326.

Cosgrove, D.J. (2016). Catalysts of plant cell wall loosening. F1000Res. **5**. F1000 Faculty Rev-119.

Cruz-Valderrama, J.E., Gómez-Maqueo, X., Salazar-Iribe, A., Zúñiga-Sánchez, E., Hernández-Barrera, A., Quezada-Rodríguez, E., and Gamboa-deBuen, A. (2019). Overview of the Role of Cell Wall DUF642 Proteins in Plant Development. Int. J. Mol. Sci. **20**:3333.

Czechowski, T., Stitt, M., Altmann, T., Udvardi, M.K., and Scheible, W.-R. (2005). Genome-Wide Identification and Testing of Superior Reference Genes for Transcript Normalization in Arabidopsis. Plant Physiol. **139**:5–17.

Deeken, R., Engelmann, J.C., Efetova, M., Czirjak, T., Müller, T., Kaiser, W.M., Tietz, O., Krischke, M., Mueller, M.J., Palme, K., et al. (2007). An Integrated View of Gene Expression and Solute Profiles of *Arabidopsis* Tumors: A Genome-Wide Approach. Plant Cell **18**:3617–3634.

Denyer, T., Ma, X., Klesen, S., Scacchi, E., Nieselt, K., and Timmermans, M.C.P. (2019). Spatiotemporal Developmental Trajectories in the Arabidopsis Root Revealed Using High-Throughput Single-Cell RNA Sequencing. Dev. Cell **48**:840–852.e5.

Feng, M., Augstein, F., Kareem, A., and Melnyk, C.W. (2024). Plant grafting: Molecular mechanisms and applications. Mol. Plant **17**:75–91.

Friedrichsen, D.M., Nemhauser, J., Muramitsu, T., Maloof, J.N., Alonso, J., Ecker, J.R., Furuya, M., and Chory, J. (2002). Three Redundant Brassinosteroid Early Response Genes Encode Putative bHLH Transcription Factors Required for Normal Growth. Genetics **162**:1445–1456.

Fucile, G., Di Biase, D., Nahal, H., La, G., Khodabandeh, S., Chen, Y., Easley, K., Christendat, D., Kelley, L., and Provart, N.J. (2011). ePlant and the 3D Data Display Initiative: Integrative Systems Biology on the World Wide Web. PLoS One **6**:e15237.

Furuya, T., Saito, M., Uchimura, H., Satake, A., Nosaki, S., Miyakawa, T., Shimadzu, S., Yamori, W., Tanokura, M., Fukuda, H., and Kondo, Y. (2021). Gene co-expression network analysis identifies BEH3 as a stabilizer of secondary vascular development in Arabidopsis. Plant Cell **33**:2618–2636.

Gao, X., Chen, J., Dai, X., Zhang, D., and Zhao, Y. (2016). An Effective Strategy for Reliably Isolating Heritable and *Cas9* -Free Arabidopsis Mutants Generated by CRISPR/Cas9-Mediated Genome Editing. Plant Physiol. **171**:1794–1800.

Garza-Caligaris, L.E., Avendaño-Vázquez, A.O., Alvarado-López, S., Zúñiga-Sánchez, E., Orozco-Segovia, A., Pérez-Ruíz, R.V., and Gamboa-deBuen, A. (2012). At3g08030 transcript: a molecular marker of seed ageing. Ann. Bot. **110**:1253–1260.

Guo, X., Wang, J., Gardner, M., Fukuda, H., Kondo, Y., Etchells, J.P., Wang, X., and Mitchum, M.G. (2017). Identification of cyst

nematode B-type CLE peptides and modulation of the vascular stem cell pathway for feeding cell formation. PLoS Pathog. **13**:e1006142.

Gutierrez, L., Mauriat, M., Guénin, S., Pelloux, J., Lefebvre, J.-F., Louvet, R., Rusterucci, C., Moritz, T., Guerineau, F., Bellini, C., and Van Wuytswinkel, O. (2008). The lack of a systematic validation of reference genes: a serious pitfall undervalued in reverse transcription-polymerase chain reaction (RT-PCR) analysis in plants. Plant Biotechnol. J. **6**:609–618.

Heyman, J., Cools, T., Vandenbussche, F., Heyndrickx, K.S., Van Leene, J., Vercauteren, I., Vanderauwera, S., Vandepoele, K., De Jaeger, G., Van Der Straeten, D., and De Veylder, L. (2013). ERF115 Controls Root Quiescent Center Cell Division and Stem Cell Replenishment. Science **342**:860–863.

Hoermayer, L., Montesinos, J.C., Marhava, P., Benková, E., Yoshida, S., and Friml, J. (2020). Wounding-induced changes in cellular pressure and localized auxin signalling spatially coordinate restorative divisions in roots. Proc. Natl. Acad. Sci. USA **117**:15322–15331.

Holzwart, E., Huerta, A.I., Glöckner, N., Garnelo Gómez, B., Wanke, F., Augustin, S., Askani, J.C., Schürholz, A.-K., Harter, K., and Wolf, S. (2018). BRI1 controls vascular cell fate in the *Arabidopsis* root through RLP44 and phytosulfokine signaling. Proc. Natl. Acad. Sci. USA **115**:11838–11843.

Ibañes, M., Fàbregas, N., Chory, J., and Caño-Delgado, A.I. (2009). Brassinosteroid signaling and auxin transport are required to establish the periodic pattern of *Arabidopsis* shoot vascular bundles. Proc. Natl. Acad. Sci. USA **106**:13630–13635.

Ichikawa, T., Nakazawa, M., Kawashima, M., Iizumi, H., Kuroda, H., Kondou, Y., Tsuhara, Y., Suzuki, K., Ishikawa, A., Seki, M., et al. (2006). The FOX hunting system: an alternative gain-of-function gene hunting technique. Plant J. **48**:974–985.

Ikeuchi, M., Iwase, A., Rymen, B., Lambolez, A., Kojima, M., Takebayashi, Y., Heyman, J., Watanabe, S., Seo, M., De Veylder, L., et al. (2017). Wounding Triggers Callus Formation via Dynamic Hormonal and Transcriptional Changes. Plant Physiol. **175**:1158–1174.

Ikeuchi, M., Rymen, B., and Sugimoto, K. (2020). How do plants transduce wound signals to induce tissue repair and organ regeneration? Curr. Opin. Plant Biol. **57**:72–77.

Ikeuchi, M., Iwase, A., Ito, T., Tanaka, H., Favero, D.S., Kawamura, A., Sakamoto, S., Wakazaki, M., Tameshige, T., Fujii, H., et al. (2022). Wound-inducible WUSCHEL-RELATED HOMEOBOX 13 is required for callus growth and organ reconnection. Plant Physiol. **188**:425–441.

Iwase, A., Mitsuda, N., Koyama, T., Hiratsu, K., Kojima, M., Arai, T., Inoue, Y., Seki, M., Sakakibara, H., Sugimoto, K., and Ohme-Takagi, M. (2011a). The AP2/ERF Transcription Factor WIND1 Controls Cell Dedifferentiation in Arabidopsis. Curr. Biol. **21**:508–514.

Iwase, A., Mitsuda, N., Koyama, T., Hiratsu, K., Kojima, M., Arai, T., Inoue, Y., Seki, M., Sakakibara, H., Sugimoto, K., and Ohme-Takagi, M. (2011b). The AP2/ERF Transcription Factor WIND1 Controls Cell Dedifferentiation in Arabidopsis. Curr. Biol. **21**:508–514.

Iwase, A., Harashima, H., Ikeuchi, M., Rymen, B., Ohnuma, M., Komaki, S., Morohashi, K., Kurata, T., Nakata, M., Ohme-Takagi, M., et al. (2017). WIND1 Promotes Shoot Regeneration through Transcriptional Activation of *ENHANCER OF SHOOT REGENERATION1* in Arabidopsis. Plant Cell **29**:54–69.

Iwase, A., Kondo, Y., Laohavisit, A., Takebayashi, A., Ikeuchi, M., Matsuoka, K., Asahina, M., Mitsuda, N., Shirasu, K., Fukuda, H., and Sugimoto, K. (2021). WIND transcription factors orchestrate wound-induced callus formation, vascular reconnection and defense response in Arabidopsis. New Phytol. **232**:734–752.

Jean-Baptiste, K., McFaline-Figueroa, J.L., Alexandre, C.M., Dorrity, M.W., Saunders, L., Bubb, K.L., Trapnell, C., Fields, S., Queitsch, C., and Cuperus, J.T. (2019). Dynamics of Gene Expression in Single Root Cells of *Arabidopsis thaliana*. Plant Cell **31**:993–1011.

Jumper, J., Evans, R., Pritzel, A., Green, T., Figurnov, M., Ronneberger, O., Tunyasuvunakool, K., Bates, R., Žídek, A., Potapenko, A., et al. (2021). Highly accurate protein structure prediction with AlphaFold. Nature **596**:583–589.

Kalmbach, L., Bourdon, M., Belevich, I., Safran, J., Lemaire, A., Heo, J.O., Otero, S., Blob, B., Pelloux, J., Jokitalo, E., and Helariutta, Y. (2023). Putative pectate lyase PLL12 and callose deposition through polar CALS7 are necessary for long-distance phloem transport in Arabidopsis. Curr. Biol. **33**:926–939.e9.

Kang, Y.H., Breda, A., and Hardtke, C.S. (2017). Brassinosteroid signaling directs formative cell divisions and protophloem differentiation in *Arabidopsis* root meristems. Development **144**:272–280.

Kilian, J., Whitehead, D., Horak, J., Wanke, D., Weinl, S., Batistic, O., D'Angelo, C., Bornberg-Bauer, E., Kudla, J., and Harter, K. (2007). The AtGenExpress global stress expression data set: protocols, evaluation and model data analysis of UV-B light, drought and cold stress responses: AtGenExpress global abiotic stress data set. Plant J. **50**:347–363.

Kim, D., Langmead, B., and Salzberg, S.L. (2015). HISAT: a fast spliced aligner with low memory requirements. Nat. Methods **12**:357–360.

Kondo, Y., Ito, T., Nakagami, H., Hirakawa, Y., Saito, M., Tamaki, T., Shirasu, K., and Fukuda, H. (2014). Plant GSK3 proteins regulate xylem cell differentiation downstream of TDIF–TDR signalling. Nat. Commun. **5**:3504.

Kondo, Y., Fujita, T., Sugiyama, M., and Fukuda, H. (2015). A Novel System for Xylem Cell Differentiation in Arabidopsis thaliana. Mol. Plant **8**:612–621.

Kondo, Y., Nurani, A.M., Saito, C., Ichihashi, Y., Saito, M., Yamazaki, K., Mitsuda, N., Ohme-Takagi, M., and Fukuda, H. (2016). Vascular Cell Induction Culture System Using Arabidopsis Leaves (VISUAL) Reveals the Sequential Differentiation of Sieve Element-Like Cells. Plant Cell **28**:1250–1262.

Krogh, A., Larsson, B., von Heijne, G., and Sonnhammer, E.L. (2001). Predicting transmembrane protein topology with a hidden markov model: application to complete genomes11Edited by F. Cohen. J. Mol. Biol. **305**:567–580.

Kubo, M., Udagawa, M., Nishikubo, N., Horiguchi, G., Yamaguchi, M., Ito, J., Mimura, T., Fukuda, H., and Demura, T. (2005). Transcription switches for protoxylem and metaxylem vessel formation. Genes Dev. **19**:1855–1860.

Kushwah, S., Banasiak, A., Nishikubo, N., Derba-Maceluch, M., Majda, M., Endo, S., Kumar, V., Gomez, L., Gorzsas, A., McQueen-Mason, S., et al. (2020). Arabidopsis *XTH4* and *XTH9* Contribute to Wood Cell Expansion and Secondary Wall Formation. Plant Physiol. **182**:1946–1965.

Lampropoulos, A., Sutikovic, Z., Wenzl, C., Maegele, I., Lohmann, J.U., and Forner, J. (2013). GreenGate - A Novel, Versatile, and Efficient Cloning System for Plant Transgenesis. PLoS One **8**:e83043.

Liu, X., Yang, H., Wang, Y., Zhu, Z., Zhang, W., and Li, J. (2020). Comparative Transcriptomic Analysis to Identify Brassinosteroid Response Genes. Plant Physiol. **184**:1072–1082.

Livak, K.J., and Schmittgen, T.D. (2001). Analysis of relative gene expression data using real-time quantitative PCR and the 2− ΔΔCT method. Methods **25**:402–408.

Lozano-Elena, F., and Caño-Delgado, A.I. (2019). Emerging roles of vascular brassinosteroid receptors of the BRI1-like family. Curr. Opin. Plant Biol. **51**:105–113.

Matosevich, R., Cohen, I., Gil-Yarom, N., Modrego, A., Friedlander-Shani, L., Verna, C., Scarpella, E., and Efroni, I. (2020). Local auxin

biosynthesis is required for root regeneration after wounding. Nat. Plants **6**:1020–1030.

Matsuoka, K., Sugawara, E., Aoki, R., Takuma, K., Terao-Morita, M., Satoh, S., and Asahina, M. (2016). Differential Cellular Control by Cotyledon-Derived Phytohormones Involved in Graft Reunion of Arabidopsis Hypocotyls. Plant Cell Physiol. **57**:2620–2631.

Melnyk, C.W. (2017a). Monitoring Vascular Regeneration and Xylem Connectivity in Arabidopsis thaliana. In Xylem, M. de Lucas and J.P. Etchhells, eds. (Springer New York), pp. 91–102.

Melnyk, C.W. (2017b). Grafting with Arabidopsis thaliana. In Plant Hormones, J. Kleine-Vehn and M. Sauer, eds. (Springer New York), pp. 9–18.

Melnyk, C.W. (2017c). Plant grafting: insights into tissue regeneration. Regeneration **4**:3–14.

Melnyk, C.W. (2017d). Connecting the plant vasculature to friend or foe. New Phytol. **213**:1611–1617.

Melnyk, C.W., and Meyerowitz, E.M. (2015). Plant grafting. Curr. Biol. **25**:R183–R188.

Melnyk, C.W., Schuster, C., Leyser, O., and Meyerowitz, E.M. (2015). A Developmental Framework for Graft Formation and Vascular Reconnection in Arabidopsis thaliana. Curr. Biol. **25**:1306–1318.

Melnyk, C.W., Gabel, A., Hardcastle, T.J., Robinson, S., Miyashima, S., Grosse, I., and Meyerowitz, E.M. (2018). Transcriptome dynamics at *Arabidopsis* graft junctions reveal an intertissue recognition mechanism that activates vascular regeneration. Proc. Natl. Acad. Sci. USA **115**:E2447–E2456.

Mi, H., and Thomas, P. (2009). PANTHER Pathway: An Ontology-Based Pathway Database Coupled with Data Analysis Tools. In Protein Networks and Pathway Analysis, Y. Nikolsky and J. Bryant, eds. (Humana Press), pp. 123–140.

Mi, H., Muruganujan, A., Casagrande, J.T., and Thomas, P.D. (2013). Large-scale gene function analysis with the PANTHER classification system. Nat. Protoc. **8**:1551–1566.

Mi, H., Muruganujan, A., Ebert, D., Huang, X., and Thomas, P.D. (2019). PANTHER version 14: more genomes, a new PANTHER GO-slim and improvements in enrichment analysis tools. Nucleic Acids Res. **47**:D419–D426.

Mi, H., Ebert, D., Muruganujan, A., Mills, C., Albou, L.-P., Mushayamaha, T., and Thomas, P.D. (2021). PANTHER version 16: a revised family classification, tree-based classification tool, enhancer regions and extensive API. Nucleic Acids Res. **49**:D394–D403.

Miedes, E., Suslov, D., Vandenbussche, F., Kenobi, K., Ivakov, A., Van Der Straeten, D., Lorences, E.P., Mellerowicz, E.J., Verbelen, J.-P., and Vissenberg, K. (2013). Xyloglucan endotransglucosylase/hydrolase (XTH) overexpression affects growth and cell wall mechanics in etiolated Arabidopsis hypocotyls. J. Exp. Bot. **64**:2481–2497.

Murashige, T., and Skoog, F. (1962). A Revised Medium for Rapid Growth and Bio Assays with Tobacco Tissue Cultures. Physiol. Plantarum **15**:473–497.

Nakamura, A., Goda, H., Shimada, Y., and Yoshida, S. (2004). Brassinosteroid Selectively Regulates *PIN* Gene Expression in Arabidopsis. Biosci. Biotechnol. Biochem. **68**:952–954.

Nanda, A.K., and Melnyk, C.W. (2018). The role of plant hormones during grafting. J. Plant Res. **131**:49–58.

Ndimba, B.K., Chivasa, S., Hamilton, J.M., Simon, W.J., and Slabas, A.R. (2003). Proteomic analysis of changes in the extracellular matrix of Arabidopsis cell suspension cultures induced by fungal elicitors. Proteomics **3**:1047–1059.

Neff, M.M., Nguyen, S.M., Malancharuvil, E.J., Fujioka, S., Noguchi, T., Seto, H., Tsubuki, M., Honda, T., Takatsuto, S., Yoshida, S., and Chory, J. (1999). *BAS1* : A gene regulating brassinosteroid levels and light responsiveness in *Arabidopsis*. Proc. Natl. Acad. Sci. USA **96**:15316–15323.

Nurani, A.M., Ozawa, Y., Furuya, T., Sakamoto, Y., Ebine, K., Matsunaga, S., Ueda, T., Fukuda, H., and Kondo, Y. (2020). Deep Imaging Analysis in VISUAL Reveals the Role of YABBY Genes in Vascular Stem Cell Fate Determination. Plant Cell Physiol. **61**:255–264.

Oh, M.-H., Honey, S.H., and Tax, F.E. (2020). The Control of Cell Expansion, Cell Division, and Vascular Development by Brassinosteroids: A Historical Perspective. Int. J. Mol. Sci. **21**:1743.

Olmo, R., Cabrera, J., Díaz-Manzano, F.E., Ruiz-Ferrer, V., Barcala, M., Ishida, T., García, A., Andrés, M.F., Ruiz-Lara, S., Verdugo, I., et al. (2020). Root-knot nematodes induce gall formation by recruiting developmental pathways of post-embryonic organogenesis and regeneration to promote transient pluripotency. New Phytol. **227**:200–215.

Palmeros-Suárez, P.A., Massange-Sánchez, J.A., Sánchez-Segura, L., Martínez-Gallardo, N.A., Espitia Rangel, E., Gómez-Leyva, J.F., and Délano-Frier, J.P. (2017). AhDGR2, an amaranth abiotic stress-induced DUF642 protein gene, modifies cell wall structure and composition and causes salt and ABA hyper-sensibility in transgenic Arabidopsis. Planta **245**:623–640.

Pan, J., Zhao, F., Zhang, G., Pan, Y., Sun, L., Bao, N., Qin, P., Chen, L., Yu, J., Zhang, Y., and Xu, L. (2019). Control of de novo root regeneration efficiency by developmental status of Arabidopsis leaf explants. Journal of Genetics and Genomics **46**:133–140.

Prát, T., Hajný, J., Grunewald, W., Vasileva, M., Molnár, G., Tejos, R., Schmid, M., Sauer, M., and Friml, J. (2018). WRKY23 is a component of the transcriptional network mediating auxin feedback on PIN polarity. PLoS Genet. **14**:e1007177.

Ramachandran, P., Wang, G., Augstein, F., de Vries, J., and Carlsbecker, A. (2018). Continuous root xylem formation and vascular acclimation to water deficit involves endodermal ABA signalling via miR165. Development **145**:dev159202.

Ryu, K.H., Huang, L., Kang, H.M., and Schiefelbein, J. (2019). Single-Cell RNA Sequencing Resolves Molecular Relationships Among Individual Plant Cells. Plant Physiol. **179**:1444–1456.

Sala, K., Karcz, J., Rypień, A., and Kurczyńska, E.U. (2019). Unmethyl-esterified homogalacturonan and extensins seal Arabidopsis graft union. BMC Plant Biol. **19**:151.

Salazar-Iribe, A., Agredano-Moreno, L.T., Zúñiga-Sánchez, E., Jiménez-Garcia, L.F., and Gamboa-deBuen, A. (2016). The cell wall DUF642 At2g41800 (TEB) protein is involved in hypocotyl cell elongation. Plant Sci. **253**:206–214.

Shahan, R., Hsu, C.-W., Nolan, T.M., Cole, B.J., Taylor, I.W., Greenstreet, L., Zhang, S., Afanassiev, A., Vlot, A.H.C., Schiebinger, G., et al. (2022). A single-cell Arabidopsis root atlas reveals developmental trajectories in wild-type and cell identity mutants. Dev. Cell **57**:543–560.e9.

Shanks, C.M., Rice, J.H., Zubo, Y., Schaller, G.E., Hewezi, T., and Kieber, J.J. (2016). The Role of Cytokinin During Infection of *Arabidopsis thaliana* by the Cyst Nematode *Heterodera schachtii*. Molecular plant-microbe interactions **29**:57–68.

Shulse, C.N., Cole, B.J., Ciobanu, D., Lin, J., Yoshinaga, Y., Gouran, M., Turco, G.M., Zhu, Y., O'Malley, R.C., Brady, S.M., and Dickel, D.E. (2019). High-Throughput Single-Cell Transcriptome Profiling of Plant Cell Types. Cell Rep. **27**:2241–2247.e4.

Siddique, S., Radakovic, Z.S., Hiltl, C., Pellegrin, C., Baum, T.J., Beasley, H., Bent, A.F., Chitambo, O., Chopra, D., Danchin, E.G.J., et al. (2022). The genome and lifestage-specific transcriptomes of a plant-parasitic nematode and its host reveal susceptibility genes involved in trans-kingdom synthesis of vitamin B5. Nat. Commun. **13**:6190.

Szakasits, D., Heinen, P., Wieczorek, K., Hofmann, J., Wagner, F., Kreil, D.P., Sykacek, P., Grundler, F.M.W., and Bohlmann, H. (2009). The transcriptome of syncytia induced by the cyst nematode *Heterodera schachtii* in Arabidopsis roots. Plant J. **57**:771–784.

Teufel, F., Almagro Armenteros, J.J., Johansen, A.R., Gíslason, M.H., Pihl, S.I., Tsirigos, K.D., Winther, O., Brunak, S., von Heijne, G., and Nielsen, H. (2022). SignalP 6.0 predicts all five types of signal peptides using protein language models. Nat. Biotechnol. **40**:1023–1025.

Carbon, S., Douglass, E., Good, B.M., Unni, D.R., Harris, N.L., Mungall, C.J., Basu, S., Chisholm, R.L., Dodson, R.J., et al.; Gene Ontology Consortium (2021). The Gene Ontology resource: enriching a GOld mine. Nucleic Acids Res. **49**:D325–D334.

Thomas, H., Van den Broeck, L., Spurney, R., Sozzani, R., and Frank, M. (2022). Gene regulatory networks for compatible versus incompatible grafts identify a role for SlWOX4 during junction formation. Plant Cell **34**:535–556.

Ursache, R., Andersen, T.G., Marhavý, P., and Geldner, N. (2018). A protocol for combining fluorescent proteins with histological stains for diverse cell wall components. Plant J. **93**:399–412.

Varadi, M., Anyango, S., Deshpande, M., Nair, S., Natassia, C., Yordanova, G., Yuan, D., Stroe, O., Wood, G., Laydon, A., et al. (2022). AlphaFold Protein Structure Database: massively expanding the structural coverage of protein-sequence space with high-accuracy models. Nucleic Acids Res. **50**:D439–D444.

Vázquez-Lobo, A., Roujol, D., Zuñiga-Sánchez, E., Albenne, C., Piñero, D., Gamboa de Buen, A., and Jamet, E. (2012). The highly conserved spermatophyte cell wall DUF642 protein family: Phylogeny and first evidence of interaction with cell wall polysaccharides in vitro. Mol. Phylogenet. Evol. **63**:510–520.

Vert, G., Nemhauser, J.L., Geldner, N., Hong, F., and Chory, J. (2005). MOLECULAR MECHANISMS OF STEROID HORMONE SIGNALING IN PLANTS. Annu. Rev. Cell Dev. Biol. **21**:177–201.

Waese, J., Fan, J., Pasha, A., Yu, H., Fucile, G., Shi, R., Cumming, M., Kelley, L.A., Sternberg, M.J., Krishnakumar, V., et al. (2017). ePlant: Visualizing and Exploring Multiple Levels of Data for Hypothesis Generation in Plant Biology. Plant Cell **29**:1806–1821.

Wendrich, J.R., Yang, B., Vandamme, N., Verstaen, K., Smet, W., Van de Velde, C., Minne, M., Wybouw, B., Mor, E., Arents, H.E., et al. (2020). Vascular transcription factors guide plant epidermal responses to limiting phosphate conditions. Science **370**:eaay4970.

Wolf, S., Mravec, J., Greiner, S., Mouille, G., and Höfte, H. (2012). Plant Cell Wall Homeostasis Is Mediated by Brassinosteroid Feedback Signaling. Curr. Biol. **22**:1732–1737.

Wolf, S., van der Does, D., Ladwig, F., Sticht, C., Kolbeck, A., Schürholz, A.-K., Augustin, S., Keinath, N., Rausch, T., Greiner, S., et al. (2014). A receptor-like protein mediates the response to pectin modification by activating brassinosteroid signaling. Proc. Natl. Acad. Sci. USA **111**:15261–15266.

Yamaguchi, Y.L., Suzuki, R., Cabrera, J., Nakagami, S., Sagara, T., Ejima, C., Sano, R., Aoki, Y., Olmo, R., Kurata, T., et al. (2017). Root-knot and cyst nematodes activate procambium-associated genes in Arabidopsis roots. Front. Plant Sci. **8**:1195.

Zhang, Y., Lee, C.-W., Wehner, N., Imdahl, F., Svetlana, V., Weiste, C., Dröge-Laser, W., and Deeken, R. (2015). Regulation of Oncogene Expression in T-DNA-Transformed Host Plant Cells. PLoS Pathog. **11**:e1004620.

Zhang, T.-Q., Xu, Z.-G., Shang, G.-D., and Wang, J.-W. (2019). A Single-Cell RNA Sequencing Profiles the Developmental Landscape of Arabidopsis Root. Mol. Plant **12**:648–660.

Zhang, A., Matsuoka, K., Kareem, A., Robert, M., Roszak, P., Blob, B., Bisht, A., De Veylder, L., Voiniciuc, C., Asahina, M., and Melnyk, C.W. (2022). Cell-wall damage activates DOF transcription factors to promote wound healing and tissue regeneration in Arabidopsis thaliana. Curr. Biol. **32**:1883–1894.e7.

Plant Communications, Volume *6*

Supplemental information

Damage activates *EXG1* and *RLP44* to suppress vascular differentiation during regeneration in *Arabidopsis*

Shamik Mazumdar, Frauke Augstein, Ai Zhang, Constance Musseau, Muhammad Shahzad Anjam, Peter Marhavy, and Charles W. Melnyk

Damage activates *EXG1* and *RLP44* to suppress vascular differentiation during regeneration in Arabidopsis

Shamik Mazumdar[1,a], Frauke Augstein[1], Ai Zhang[1,b], Constance Musseau[1,c], Muhammad Shahzad Anjam[2], Peter Marhavy[2], Charles W. Melnyk[1*]

1 - Department of Plant Biology, Linnean Center for Plant Biology, Swedish University of Agricultural Sciences, Almas allé 5, 756 51, Uppsala, Sweden

2 - Umea Plant Science Centre (UPSC), Department of Forest Genetics and Plant Physiology, Swedish University of Agricultural Sciences (SLU), 90183 Umea, Sweden

a - Present address: Department of Molecular Sciences, Linnean Center for Plant Biology, Swedish University of Agricultural Sciences, Almas allé 5, 756 51, Uppsala, Sweden

b - present address: College of Life Sciences, Northwest A&F University, No.3 Taicheng Road, Yangling, Shaanxi, China, 712100

c - Present address: Syngenta Seeds, Westeinde 62, 1601 BK Enkhuizen, Netherlands

* **Corresponding Author:** Charles W. Melnyk, email: charles.melnyk@slu.se, Department of Plant Biology, Linnean Center for Plant Biology, Swedish University of Agricultural Sciences, Almas allé 5, 756 51, Uppsala, Sweden

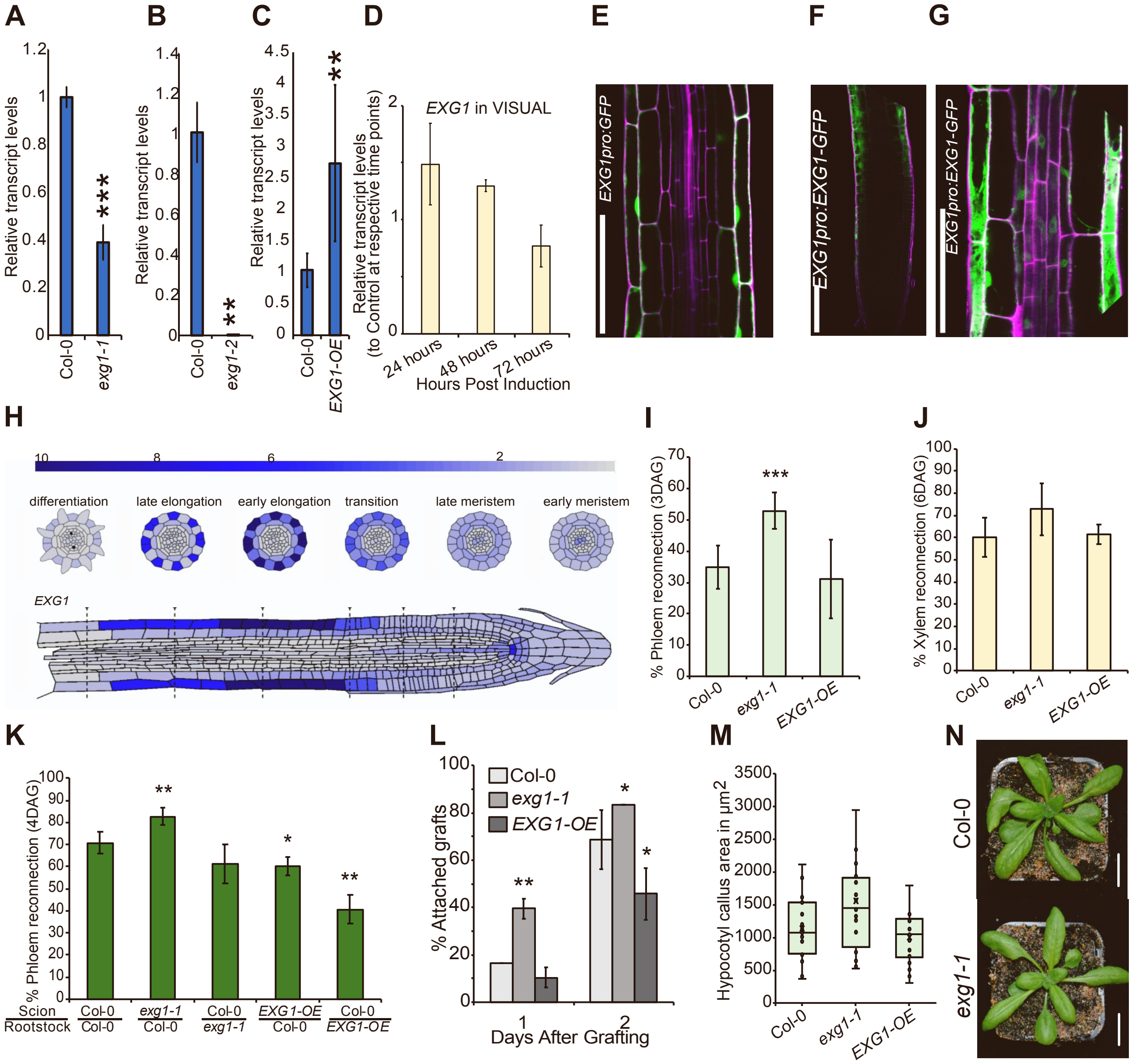

Supplemental Figure 1. *EXG1* expression levels, phenotypes. A-B. Expression of *EXG1* in *exg1-1* and *exg1-2* T-DNA insertion lines. C. Expression of *EXG1* in FOX hunting line *EXG1-OE*. D. Transcript levels of *EXG1* during VISUAL induction. E-F. *EXG1pro:GFP* fluorescence at the transition zone in the roots and at the root tip. G. *EXG1pro:EXG1-GFP* fluorescence at the transition zone in the roots. Cell walls stained by PI (magenta). Scale bar represents 100 µm. H. *EXG1* expression pattern in different cell layers of the primary root as generated from Root Cell Atlas (https://rootcellatlas.org/)Ryu et al., 2019; Denyer et al., 2019; Shulse et al., 2019; Jean-Baptiste et al., 2019; Wendrich et al., 2020; Shahan et al., 2022). I-J. Reconnection percentage of phloem (3 days after grafting – DAG) and xylem (6 DAG). The mean ± SD of 4-5 experiments is shown. K. Reconnection percentage of phloem (4DAG) during *EXG1* heterografting. The mean ± SD of 4 experiments is shown. L. Percentage of attached grafts at 1 DAG and 2 DAG. The mean ± SD of 4 experiments is shown. M. Hypocotyl explant callus areas of Col-0, *exg1-1* and *EXG1-OE*. Dots represent individual samples. N. Col-0 and exg1-1 plants in pots. Scale bar represents 1 cm. Asterisks indicate significant differences compared to Col-0. For transcript levels significance was calculate using Student's t-test ** p <0.01, *** p <0.001. For grafting and attachment assays significance was calculated by pairwise t-tests with Benjamini-Hochberg adjustment, *p<0.05, **p<0.01, ***p<0.001.

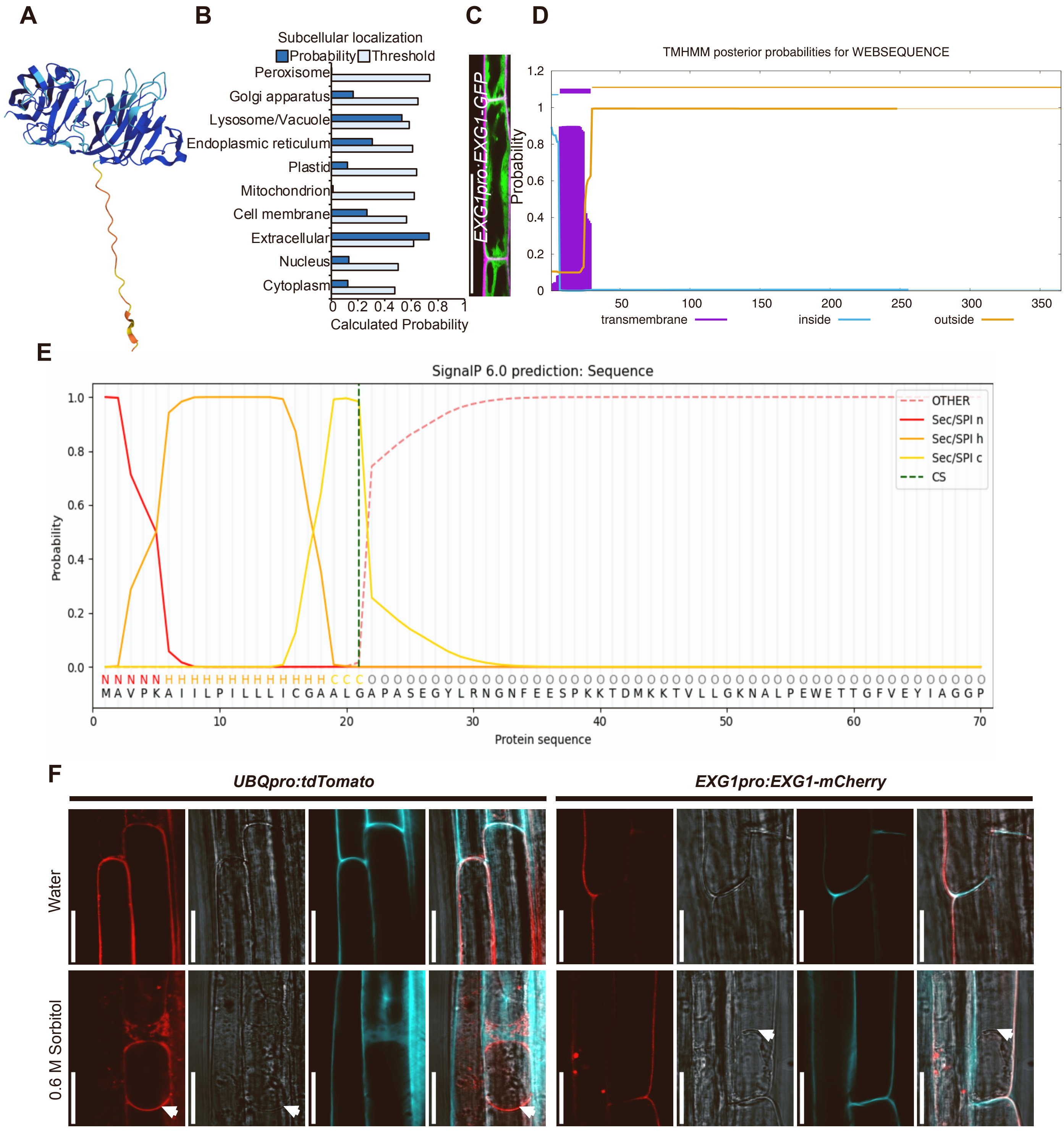

Supplemental Figure 2. ***EXG1*** **protein predictions and subcellular localiations.** A. Predicted protein structure of EXG1 using AlphaFold 2.0 (Jumper et al., 2021; Varadi et al., 2021). B. Threshold and calculated probability of subcellular localization of the EXG1 protein, predicted using DeepLoc2.0 (Thumuluri et al., 2022). C. *EXG1pro:EXG1-GFP* fluorescence in root tip epidermal cell. Cell walls stained by PI (magenta). Scale bar represents 50 µm. D. Transmembrane domain prediction using TMHMM (Krogh et al., 2001). E. Signal sequence prediction using Signal IP6.0 (Teufel et al., 2022). (F) Images showing plasmolysis of *EXG1pro:EXG1-mCherry* and *UBQpro:tdTomato* (in red), cell wall marked by Calcofluor white (cyan) in water and 0.6M sorbitol. White arrows indicate plasma membrane. Scale bars represent 20 µm.

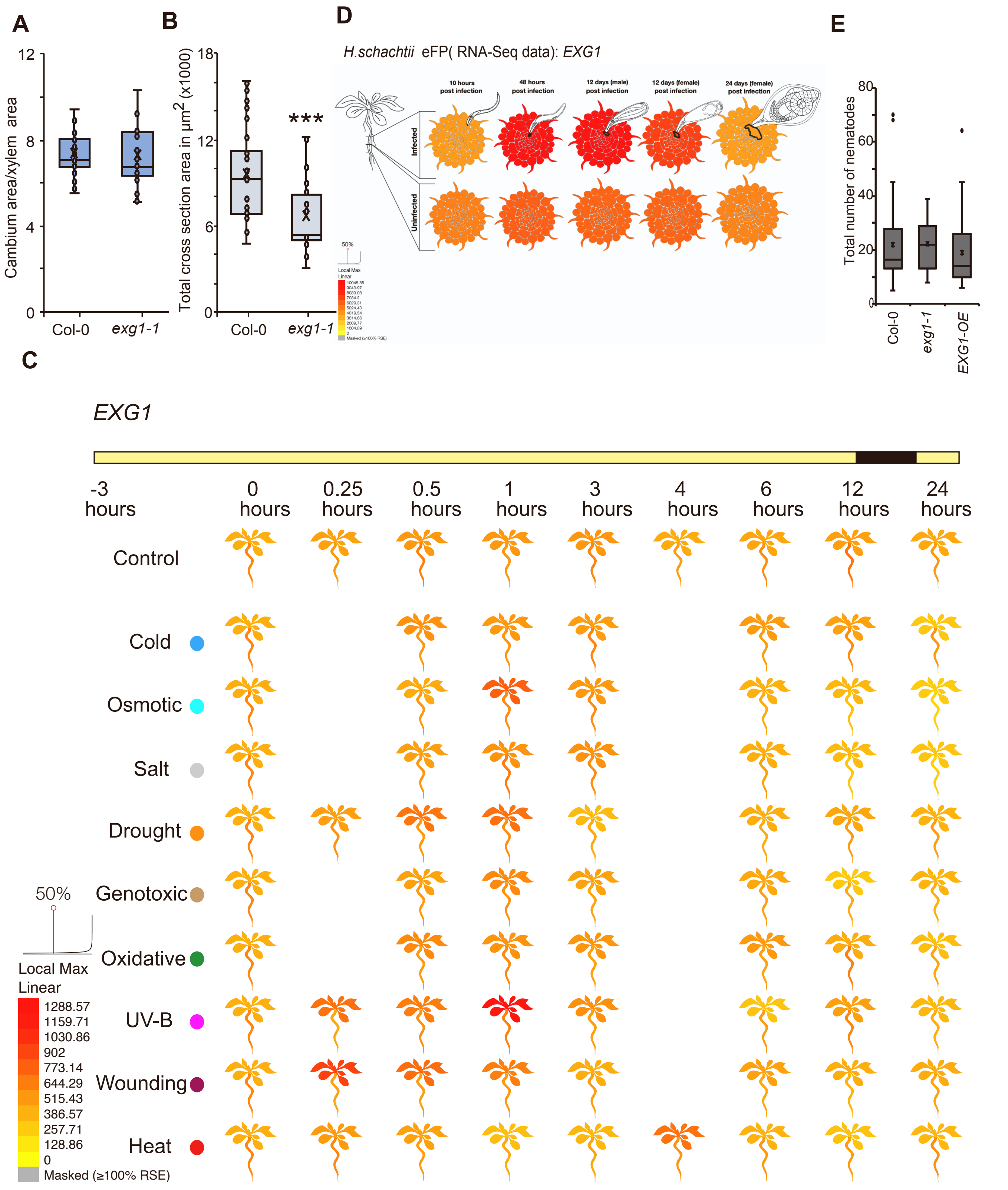

Supplemental Figure 3. *In silico* expression profiles of *EXG1* during stress, nematode infection. A. Cambium:xylem area quantifications. Dots represent individual samples. B. Total cross section area quantifications. Dots represent individual samples. C. *EXG1* expression during different abiotic stresses, eFP Browser profile (Kilian et al., 2007; Fucile et al., 2011). D. *EXG1* expression during *H. schachtii* infection. Data from ePlant (Fucile et al., 2011; Bar Toronto). E. Total number of nematodes per plant in Col-0, *exg1-1* and *EXG1-OE*. Dots indicate individual samples. Asterisks indicate statistical significance. For cross sections significance calculated by Wilcoxon's test, *** p<0.001.

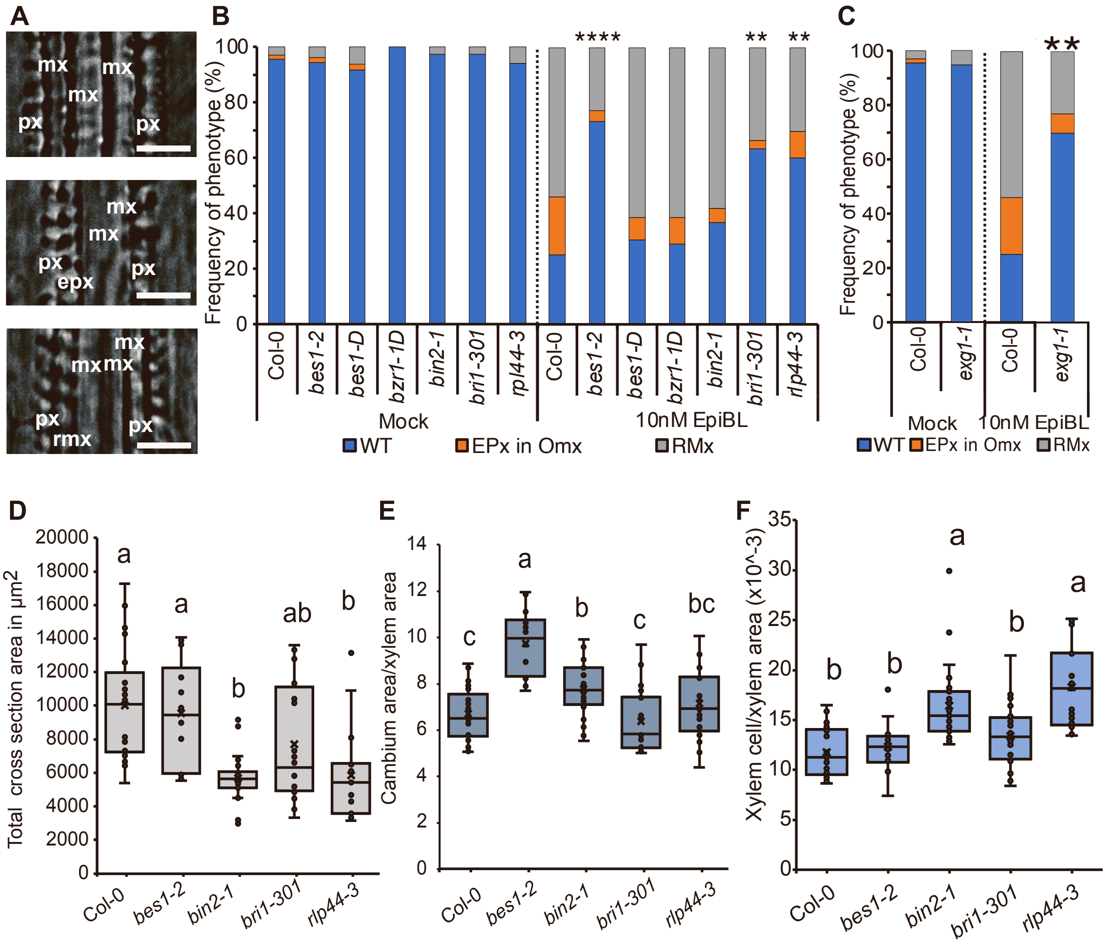

Supplemental Figure 4. Brassinosteroid mutants and *RLP44* in non-wounded conditions. A. Images showing primary root xylem phenotypes including protoxylem (px), metaxylem (mx), extra protoxylem (epx), reticulate metaxylem (rmx). Scale bar represents 200µM. B. Xylem phenotypes under mock and exogenous epiBL treatments: wild type (WT), extra protoxylem in outer metaxylem (EPx in Omx) or reticulate metaxylem (RMx) (n≥32). C. Xylem phenotypes of under mock and exogenous epiBL treatment: wild type (WT), extra protoxylem in outer metaxylem (EPx in Omx) or reticulate metaxylem (RMx) (n=50). D. Total cross section area comparisons. Dots represent samples. E. Cambium:xylem area comparisons. Dots represent samples. F. Xylem cell/ unit xylem area comparisons. Dots represent samples. Asterisks indicate statistical significance. For xylem phenotype significance was calculated by Fisher's exact test with Benjamini-Hochberg adjustment, **p<0.01, ****p<0.0001. For cross section significance was calculated by One-way ANOVA, with Tukey's post-hoc test. Compact letters indicate significant differences.

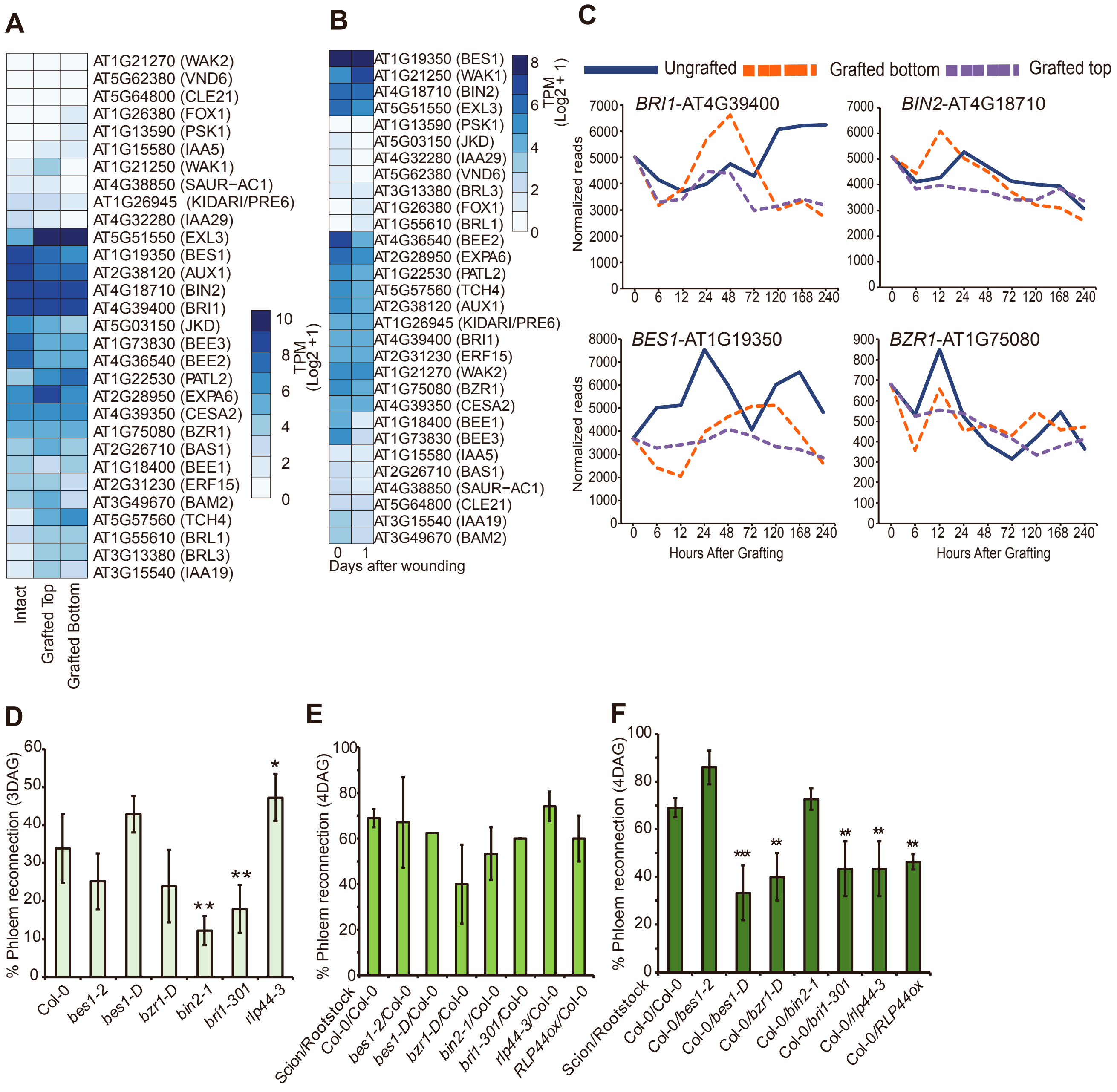

Supplemental Figure 5: BR responses and requirements at the graft junction. A. Heat map of the expression levels of brassinosteroid responsive genes at 24 hours after grafting (Zhang et al., 2022). B. Heat map of the expression levels of brassinosteroid responsive genes in petioles after wounding (Pan et al., 2019). C. Expression profile of brassinosteroid induced genes during graft formation (Melnyk et al. 2018). D. Reconnection percentage of phloem (3DAG). Mean ± SD of 4 experiments is shown. E-F. Reconnection percentage of phloem (4DAG) during heterografting. Mean ± SD of 3 experiments is shown. Asterisks indicate statistical significance. For grafting assays significance calculated by pairwise t-tests with Benjamini-Hochberg adjustment *p<0.05, ** p<0.01, *** p<0.001.

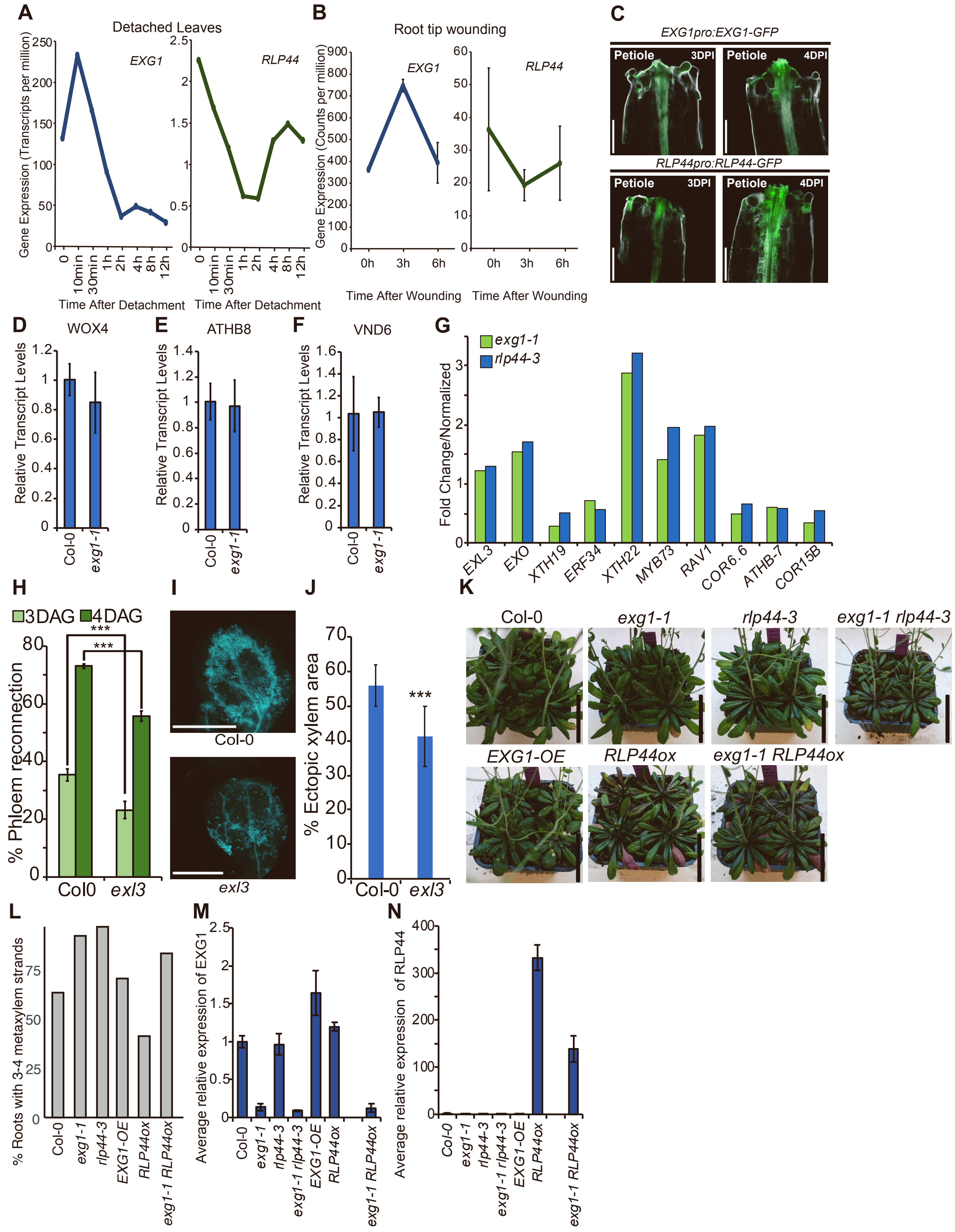

Supplemental Figure 6. *EXG1* is stress responsive and shares common downstream targets with *RLP44*. A. Expression of *EXG1* and *RLP44* in 12-day old, detached leaves of seedlings (Zhang et al., 2019) B. Expression of *EXG1* and *RLP44* in wounded root tips (Matosevich et al., 2020). C. *EXG1pro:EXG1-GFP* and *RLP44pro:RLP44-GFP* fusion protein fluorescence during petiole callus formation at 3 and 4 days post induction. Cell walls stained by Calcoflour white (grey). Scale bars represent 200 μm. D. Relative transcript levels of WOX4 in *exg1-1* compared to WT Col-0. E. Relative transcript levels of ATHB8 in *exg1-1* compared to WT Col-0. F. Relative transcript levels of VND6 in *exg1-1* compared to WT Col-0. G. Bar plot showing Fold change of a subset of genes differentially expressed in both *exg1-1* and *rlp44-3.* H. Reconnection percentage of phloem 3 and 4DAG. The mean ± SD of 3 experiments is shown. Asterisks indicate significant differences compared to Col-0. *** $p<0.001$, Student's t-test. I. VISUAL assay images of ectopic xylem formation. Scale bar represents 1 mm. J. % Ectopic xylem area quantifications. K. Images of mature plants. Scale bars represent 1cm. L. Metaxylem strand percentage. M. qPCR for average transcript level of EXG1. N. qPCR for average transcript level of RLP44. Asterisks indicate significant differences. For grafting assays significance was calculated using Student's t-test, ***$p<0.001$. For VISUAL assays significance was calculated using Wilcoxon's test, ***$p<0.001$.

Supplemental Table 1: List of lines used and generated in this study

AGI code	Name	Line	Mutant	Source	Donated by
AT3G08030	*EXG1*	*exg1-1*	SALK_011516C	This Study	NASC
AT3G08030	*EXG1*	*exg1-2*	SALK_119379C	This Study	NASC
AT3G08030	*EXG1*	*EXG1-OE*	-	(Ichikawa et al. 2006)	RIKEN BRC
AT3G08030	*EXG1*	*EXG1pro:GFP*	-	This Study	-
AT3G08030	*EXG1*	*EXG1pro:EXG1-GFP*		This Study	-
AT3G08030	*EXG1*	*EXG1pro:EXG1-mCherry*		This Study	-
-	-	*UBQpro:tdTomato*		(Segonzac et al. 2012)	Elliot Meyerowitz
AT3G49750	*RLP44*	*rlp44-3*	SAIL_596_E12	(Wolf et al. 2014)	Sebastian Wolf
AT3G49750	*RLP44*	*RLP44ox*	-	(Wolf et al. 2014)	
AT4G32880	*ATHB8*	*athb8-12*	-	(Prigge et al. 2005)	Annelie Carlsbecker
AT5G51550	*EXL3*	*exl3*	SALK_070475C	This Study	NASC
AT3G22600	*LTPG5*	*ltpg5*	SALK_007462C	(Edstam and Edqvist 2014)	NASC
AT5G10930	*CIPK5*	*cipk5*	SALK_063555C	(Förster et al. 2019)	NASC
AT4G24130	*SVB5*	*svb5*	SALK_006910C	(Hussain et al. 2021)	NASC
AT4G39400	*BRI1*	*bri1-301*		(Li and Nam 2002)	Sebastian Wolf
AT4G18710	*BIN2*	*bin2-1*		(Li et al. 2001)	
AT1G19350	*BES1*	*bes1-2*	WiscDsLox246D02	(Lachowiec et al. 2013)	Yuki Kondo
AT1G19350	*BES1*	*bes1-D*		(Yin et al. 2002)	
AT1G75080	*BZR1*	*bzr1-D*		(Wang et al. 2002)	

Supplemental Table 2: Primers used for genotyping

Name	Sequence	Reason
EXG1-F1	CAAAGACGACAAAATTCCCAC	Genotyping *EXG1-1*
EXG1-R1	CGTGAGTGCGTAGAGAGAACC	
EXG1-F2	CACTAACCAAGGAAAACTCATTCTC	Genotyping *EXG1-2*
EXG1-R2	CAAGCTCTTTAATGGCGACAG	
Exl3-F1	GCAATCATTAGGCAAAAGCTG	Genotyping *exl3*
Exl3-R1	TCAGCCGTTGGATCTAAACAC	
CIPK5-F1	GGAAAGTACGAGATGGGAAGG	Genotyping *cipk5*
CIPK5-R1	TGTTCATCTCCTTCGTAACCG	
LTPG5-F1	TGGGATGCGTACATGTGTATG	Genotyping *ltpg5*
LTPG5-R1	ATGTAGTTGAGACACGGCGAC	
SVB5-F1	TTGCTTCATCCAAACGTAACC	Genotyping *svb5*
SVB5-R1	AAGGGCTTTGGACTTTACCTG	
RLP44-3-F1	ATTCCACTCCCAAGTCCAATC	Genotyping *rlp44-3*
RLP44-3-R1	AATGGATGGCATGATTAGGATC	
SALK_LB1	ATTTTGCCGATTTCGGAAC	LBb1.3 SALK pROK2
SAIL_LB1	GCCTTTTCAGAAATGGATAAATAGCCTTGCTTCC	LB1 for SAIL lines C/418-451 of pCSA110-pDAP101_T-DNAs
pDs-Lox_LB1	AACGTCCGCAATGTGTTATTAAGTTGTC	WiscDsLox T-DNA LB primer P745

Supplemental Table 3: Primers used for expression analysis

Name	Sequence	Reason
EXG1_qP-F	GCTCCTGCTTCTGAAGGTTATC	qPCR primers for EXG1
EXG1_qP-R	AACCGGTGGTTTCCCATTCG	
EXL3-qP-F1	CAAGATTTTTGCGGCCAGGT	qPCR primers for EXL3
EXL3-qP-R1	AAGCACAAACTCCGGGACAA	
CIPK5-qP-F1	CGCCGAAATTCTTCAACGCT	qPCR primers for CIPK5
CIPK5-qP-R1	GACCGTGACGTAAACACCGA	
LTPG5-qP-F1	CTTGGTCTGCCTAGGGCTTG	qPCR primers for LTPG5
LTPG5-qP-R1	CCTGGTCCTGAAGAGTTTGG	
SVB5-qP-F1	ATGACAGGAGTGAAGAGTAAGCAG	qPCR primers for SVB5
SVB5-qP-R1	CGGTAACGGGGAAAGACCTG	

Supplemental Table 4: Primers used for Cloning

Name	Sequence	Cloning module	Reason
EXG1-proF	AACAGGTCTCAACCTCACCGATGGTGACATTG	Greengate	*EXG1* promoter
EXG1-proR	AACAGGTCTCATGTTTGTCTCTGTTGTTCTTCC	Greengate	
RbcsT-F	AACAGGTCTCACTGCAGAGCTTTCGTTCGTATC	Greengate	Pea Rbcs9-E terminator
RbcsT-R	ACAAGGTCTCATAGTGTTGTCAATCAATTGGC	Greengate	
GFP-CDS-F	AACAGGTCTCAGGCTATGGTGAGCAAGGGCG	Greengate	GFP coding sequence
GFP-CDS-R	ACAAGGTCTCACTGACTACTTGTACAGCTCGTCC	Greengate	

References

Edstam MM and Edqvist J. Involvement of GPI-anchored lipid transfer proteins in the development of seed coats and pollen in *Arabidopsis thaliana*. Physiol Plantarum. 2014:**152**(1):32–42. https://doi.org/10.1111/ppl.12156

Förster S, Schmidt LK, Kopic E, Anschütz U, Huang S, Schlücking K, Köster P, Waadt R, Larrieu A, Batistič O, et al. Wounding-Induced Stomatal Closure Requires Jasmonate-Mediated Activation of GORK K+ Channels by a Ca2+ Sensor-Kinase CBL1-CIPK5 Complex. Developmental Cell. 2019:**48**(1):87-99.e6. https://doi.org/10.1016/j.devcel.2018.11.014

Hussain S, Zhang N, Wang W, Ahmed S, Cheng Y, Chen S, Wang X, Wang Y, Hu X, Wang T, et al. Involvement of ABA Responsive SVB Genes in the Regulation of Trichome Formation in Arabidopsis. IJMS. 2021:**22**(13):6790. https://doi.org/10.3390/ijms22136790

Ichikawa T, Nakazawa M, Kawashima M, Iizumi H, Kuroda H, Kondou Y, Tsuhara Y, Suzuki K, Ishikawa A, Seki M, et al. The FOX hunting system: an alternative gain-of-function gene hunting technique. The Plant Journal. 2006:**48**(6):974–985. https://doi.org/10.1111/j.1365-313X.2006.02924.x

Lachowiec J, Lemus T, Thomas JH, Murphy PJM, Nemhauser JL, and Queitsch C. The Protein Chaperone HSP90 Can Facilitate the Divergence of Gene Duplicates. Genetics. 2013:**193**(4):1269–1277. https://doi.org/10.1534/genetics.112.148098

Li J and Nam KH. Regulation of Brassinosteroid Signaling by a GSK3/SHAGGY-Like Kinase. Science. 2002:**295**(5558):1299–1301. https://doi.org/10.1126/science.1065769

Li J, Nam KH, Vafeados D, and Chory J. *BIN2* , a New Brassinosteroid-Insensitive Locus in Arabidopsis. Plant Physiology. 2001:**127**(1):14–22. https://doi.org/10.1104/pp.127.1.14

Prigge MJ, Otsuga D, Alonso JM, Ecker JR, Drews GN, and Clark SE. Class III Homeodomain-Leucine Zipper Gene Family Members Have Overlapping, Antagonistic, and Distinct Roles in Arabidopsis Development. The Plant Cell. 2005:**17**(1):61–76. https://doi.org/10.1105/tpc.104.026161

Segonzac C, Nimchuk ZL, Beck M, Tarr PT, Robatzek S, Meyerowitz EM, and Zipfel C. The Shoot Apical Meristem Regulatory Peptide CLV3 Does Not Activate Innate Immunity. Plant Cell. 2012:**24**(8):3186–3192. https://doi.org/10.1105/tpc.111.091264

Wang Z-Y, Nakano T, Gendron J, He J, Chen M, Vafeados D, Yang Y, Fujioka S, Yoshida S, Asami T, et al. Nuclear-Localized BZR1 Mediates Brassinosteroid-Induced Growth and Feedback Suppression of Brassinosteroid Biosynthesis. Developmental Cell. 2002:**2**(4):505–513. https://doi.org/10.1016/S1534-5807(02)00153-3

Wolf S, van der Does D, Ladwig F, Sticht C, Kolbeck A, Schürholz A-K, Augustin S, Keinath N, Rausch T, Greiner S, et al. A receptor-like protein mediates the response to pectin modification by activating brassinosteroid signaling. Proc Natl Acad Sci USA. 2014:**111**(42):15261–15266. https://doi.org/10.1073/pnas.1322979111

Yin Y, Wang Z-Y, Mora-Garcia S, Li J, Yoshida S, Asami T, and Chory J. BES1 Accumulates in the Nucleus in Response to Brassinosteroids to Regulate Gene Expression and Promote Stem Elongation. Cell. 2002:**109**(2):181–191. https://doi.org/10.1016/S0092-8674(02)00721-3
